# Brain endothelial spheroids and cortical organoids reveal the impact of *Toxoplasma gondii* lineage and host-phagocyte-pathogen interactions on colonization

**DOI:** 10.1007/s00018-025-06035-7

**Published:** 2026-01-29

**Authors:** Matias E. Rodriguez, Elena Afanaseva, Ali Hassan, Felix Harryson-Oliveberg, Antonio Barragan

**Affiliations:** Department of Molecular Biosciences, The Wenner-Gren Institute, Stockholm University, Stockholm, Sweden

**Keywords:** (MeSH): Three-dimensional cell culture, Host-pathogen, Central nervous system protozoal infections, Intracellular parasitism, Blood-brain barrier, leukocyte migration

## Abstract

*Toxoplasma gondii* chronically infects the central nervous system (CNS), but the mechanisms enabling its traversal of the blood-brain barrier (BBB) remain unclear. Here, we investigated BBB penetration using brain endothelial spheroids and cerebral tissue-derived organoids that recapitulate three-dimensional barrier features. We show that *T. gondii* tachyzoites efficiently colonize spheroids, without detectable barrier disruption or obligatory parasite replication. Following direct transmigration, tachyzoites invaded and replicated within deeper cell layers. Type I strains (RH, CPS) exhibited higher colonization efficiency than type II strains (PRU, ME49), independent of replication. In contrast, when spheroids were exposed to *T. gondii*-infected dendritic cells (DCs), both strain types were transported similarly into deep cellular layers. Infected DCs adopted an amoeboid-like migratory phenotype that facilitated parasite transport and subsequent dissemination after egress. Colonization was attenuated by ICAM-1 blockade or heparin treatment, while the parasite effector GRA15, despite modulating DC-endothelial adhesion, did not significantly impact intratissue migration. In contrast, deletion of the effector TgWIP markedly reduced the number of infected DCs entering the spheroids. Similar colonization dynamics were observed in murine cerebral organoids. Collectively, these findings highlight spheroid and organoid models as robust systems for uncovering the cellular and molecular mechanisms underlying *T. gondii* BBB traversal and CNS colonization.

**Supplementary Information:**

The online version contains supplementary material available at 10.1007/s00018-025-06035-7.

## Introduction

The blood-brain barrier (BBB), located within the cerebral microvasculature, serves as a protective shield for the vertebrate central nervous system (CNS), guarding it against most infectious threats [1, 2]. This barrier is composed of a highly specialized endothelial layer that has low permeability and expresses intercellular tight junction (TJ) proteins, which limit the movement of hydrophilic molecules and control cell migration across the endothelial layer [3, 4]. Beneath these endothelial cells, the basal lamina, pericytes, and astrocyte “end-feet” further contribute to the restricted permeability of the neurovascular unit (NVU) wall. Combined, these layers create a highly restrictive tissue barrier with a thickness of less than 10 μm [5–7].

Spheroids and organoids are three-dimensional cellular models used in biological research to better replicate the architecture and microenvironment of tissues in living organisms [8]. Unlike traditional two-dimensional cultures, these multicellular constructs enable cells to grow in all directions, promoting more physiological cell-cell and cell-matrix interactions. Although widely used in cancer research [9], spheroids and organoids are increasingly being applied in infection biology, providing a more accurate platform for studying infection dynamics and evaluating antimicrobial therapies [10].

CNS infections rank among the most devastating infectious diseases worldwide and are often associated with severely limited therapeutic options [11]. The Apicomplexan parasite *Toxoplasma gondii* infects a wide range of warm-blooded vertebrates, with approximately one-third of the global human population estimated to be chronically infected [12]. After entering through the intestine, *T. gondii* disseminates systemically via the circulatory systems, quickly establishing a latent infection in the CNS [13]. Although chronic infection is typically asymptomatic, reactivation or acute infection can cause life-threatening encephalitis in immunocompromised individuals and severe neurological disorders in neonates [14]. Three clonal *T. gondii* lineages (type I, II, III) predominate in human and animal infections across Europe and North America [15].

*T. gondii* tachyzoites are obligate intracellular and use gliding motility to actively invade cells where they replicate in a parasitophorous vacuole (PV) [16]. Gliding motility also facilitates transmigration of tachyzoites across polarized endothelial monolayers in vitro [17, 18] and likely provides an effective mechanism of propulsion in the microenvironment inside tissues [19]. In their journey to form chronic cysts, the tachyzoites cross the cellular brain barriers to establish latent infection in the CNS [20]. *T. gondii* also leverages the trafficking of mononuclear phagocytes for its systemic spread [21, 22]. Upon active invasion, *T. gondii* induces dendritic cells (DCs) and other mononuclear phagocytes to migrate by activating non-canonical GABAergic signaling and MAP kinase pathways [23–26]. This migration involves secreted parasite effectors [27–29] and the chemotactic activation of infected phagocytes [30].

In recent years, various pathways and mechanisms for *T. gondii* dissemination and brain invasion have been proposed. However, the relative contributions and significance of these potential pathways across the BBB are not fully understood [20]. To gain new insights into the initial mechanisms of BBB passage, we investigated *T. gondii* colonization in brain endothelial spheroids and cortical tissue organoids.

## Results

### *T. gondii* tachyzoites cross TJ-expressing endothelium and reach deep cellular layers of endothelial spheroids, with variations between strains

**Fig. 1:**
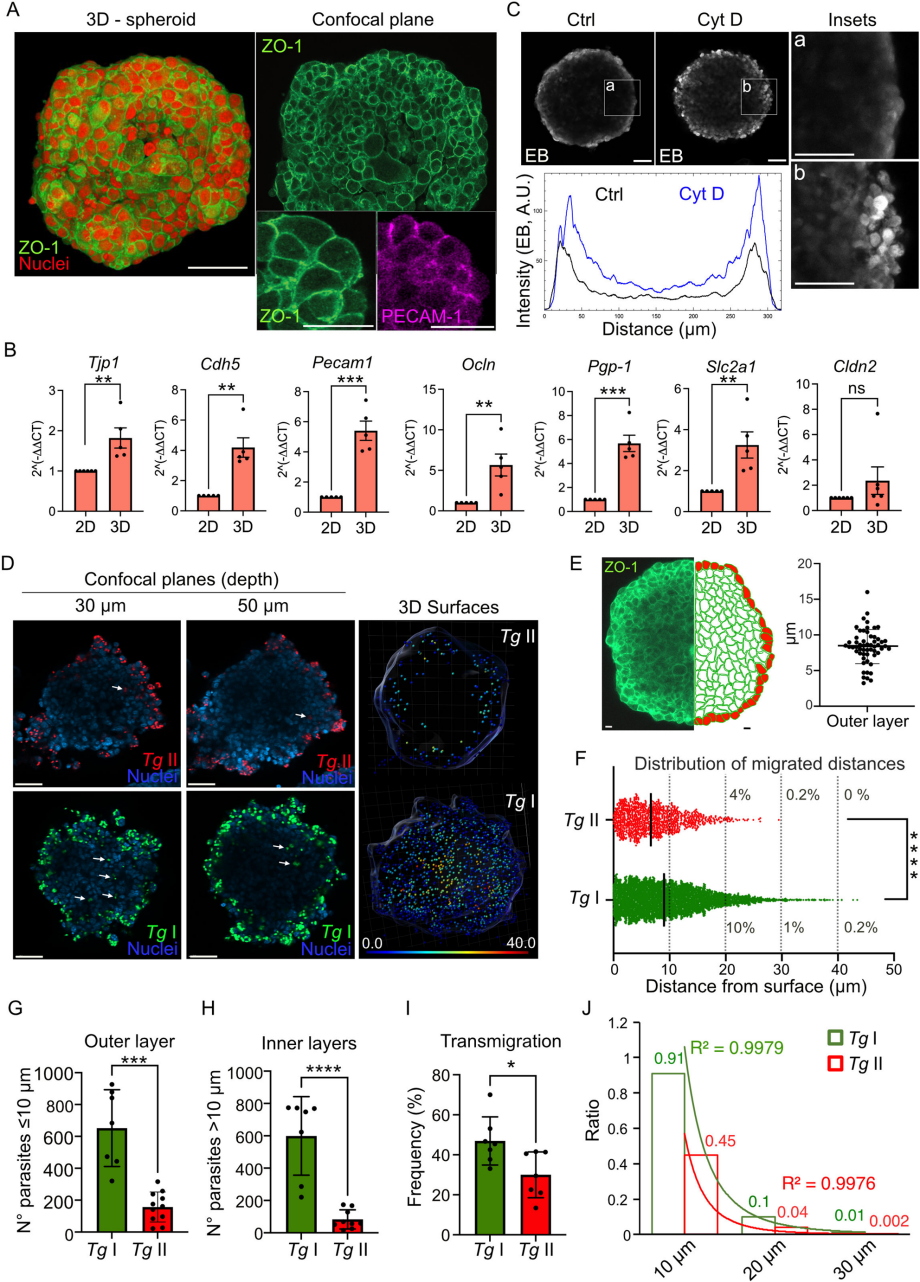
Characteristics of spheroids and colonization by *T. gondii*. **A**. 3D projection and confocal plane micrographs show the localization of cell nuclei (DAPI^+^, red), TJ marker ZO-1 (green) and cell adhesion molecule CD31/PECAM1 (inset, magenta) in a bEnd.3 cell spheroid. Spheroids were generated and stained with antibodies as indicated under Materials and Methods. Scale bars, 50 μm; inset scale bars, 20 μm. **B**. Relative mRNA expression (qPCR) of brain endothelial markers *Tjp1* (ZO-1), *Cdh5* (Cadherin-5), *Pecam1* (PECAM-1), *Ocln* (Occludin), *Pgp-1* (P-glycoprotein 1), *Slc2a1* (GLUT-1) and *Cldn2* (Claudin-2) in bEnd.3 monolayers (2D) compared with bEnd.3 spheroids (3D) at 4 and 3 d post-seeding, respectively. Data are expressed as mean (± SEM) from five independent experiments (*n* = 5) **C**. Confocal micrographs show fluorescence signal of the permeability marker Evans blue (EB, grey) in bEnd.3 spheroids following incubation with EB and depolarizing agent cytochalasin D (Cyt D, 20 ng/ml) or mock treatment (Ctrl) for 1 h. Insets (a, b) show magnified views of the surface cell layers. Scale bars, 50 μm. Graph shows the relative fluorescence intensity (arbitrary units, A. U.) of EB across the spheroid midline. **D**. Confocal micrographs (left panels) show localization of type I RH (*Tg* I, GFP^+^, green) and type II ME49 (*Tg* II, RFP^+^, red) tachyzoites in relation to nuclei (DAPI^+^, blue) at 30 and 50 μm depth inside spheroids following incubation with 5 × 10^4^ cfu *Tg*/spheroid for 24 h. Arrowheads indicate tachyzoites located at 30–50 μm from the surface of the spheroid. Scale bar: 50 μm. 3D surface analysis (right panels) from confocal stacks (0–70 μm depth planes) show the 3D distribution of *Tg* I and *Tg* II tachyzoites, respectively, measured as indicated under Materials and Methods and detailed in Fig. S2. The 3D maps show the surface of the spheroids and the tachyzoites, represented by the dots and color-coded by their distance from the nearest surface, according to color-coded scale (µm). **E.** Confocal micrograph (left) shows ZO-1 signal (green) at 50 μm depth in a bEnd.3 spheroid. Cartoon (right) illustrates cell layers with the outer cell layer highlighted in red color. Cell layers below the outer layer were defined as inner layers (green). Scale bars, 10 μm. Graph shows the height (median, 25th and 75th percentiles) of the outer layer of cells in spheroid (*n* = 54 cells). **F**. Graph shows distance from the nearest spheroid surface (µm) of individual *Tg* I and *Tg* II tachyzoites (dots) from the 3D maps. Dotted lines indicate depth of 10, 20, 30 and 40 μm and relative number of tachyzoites (%) reaching that depth. Bars indicate mean migration depth (*n* = 4.810 (*Tg* I) to 1.085 (*Tg* II) tachyzoites from 7 (*Tg* I) and 12 (*Tg* II) spheroids). **G**,** H**. Graphs show the absolute numbers (mean ± SEM) of type I and II *T. gondii* tachyzoites per spheroid located at ≤ 10 μm (G, outer layer) and > 10 μm (H, inner layers) at 24 hpi. (*n* = 8–12 spheroids). **I.** Graph shows the percentage (mean ± SEM) of tachyzoites per spheroid penetrating > 10 μm related to total number of tachyzoites in spheroid (transmigration) at 24 hpi (*n* = 7 (*Tg* I) and 12 (*Tg* II) spheroids). **J**. R-squared regression (R^2^) analysis show ratio of tachyzoites (*Tg* I, green; *Tg* II, red) penetrating the spheroids at indicated depths, related to total numbers of tachyzoites in the spheroids (1). Best-fit curve shows decay of penetration in relation to depth within the spheroid (*n* = 2.773 (*Tg* II) and 8.759 (*Tg* I) tachyzoites from 11 and 7 spheroids, respectively) All data are from 3 independent experiments, unless differently stated. Statistical analyses: (F) 2-tailed Mann-Whitney U-test; (B, G, H, I) 2-tailed unpaired Student’s *t*-test. * *p* < 0.05; ** *p* < 0.01; *** *p* < 0.001; **** *p* < 0.0001; ns: non-significant

Previous studies of *T. gondii* transmigration across BBB models have been carried out using endothelial monolayers [17]. Spheroids offer advantages, as they typically better mimic the polarized organization of cells within biological barriers, with stable expression of TJs [8]. We first evaluated a protocol for cellular spheroid formation [31] using the murine brain endothelial cell line bEnd.3 [32]. Under the applied conditions, bEnd.3 cells consistently formed compact spheroidal structures of ~ 400 μm diameter within 3 days, exhibiting expression of TJ and endothelial markers (Fig. 1A, Fig. S1A). Transcriptional expression levels of these markers were elevated compared to bEnd.3 monolayers (Fig. 1B, Fig. S1B). In addition, spheroids displayed restricted permeability to macromolecules (Evans blue (EB)/serum albumin complexes) (Fig. 1C, Fig. S1C), indicating cellular polarization.

Next, spheroids were challenged with freshly egressed type II (ME49-RFP) or type I (RH-GFP) *T. gondii* tachyzoites. Interestingly, tachyzoites readily localized inside the spheroids **(**Fig. 1D**)**. Euclidian distances of tachyzoites to the nearest spheroid surface were measured as detailed under Materials and Methods (Fig. S2). Based on confocal microscopy analyses, we defined the outer cell layer of the spheroids as bEnd.3 cells located within 10 μm of the surface and, the inner layers as cell layers located deeper than 10 μm (Fig. 1E). Upon *T. gondii* challenge, we found tachyzoite vacuoles within the outer endothelial layer (≤ 10 μm) and also in inner cell layers (> 10 μm), the latter implying crossing layers of TJ (ZO-1)-expressing cells (Fig. 1E, F; Movies S1, S2, S3). Significantly higher absolute numbers of type I tachyzoites (RH) were retrieved in the outer cell layer compared with type II (ME49) (Fig. 1G), indicating a superior cell invasion ability by type I tachyzoites. Similarly, significantly higher absolute numbers of type I tachyzoites were retrieved in inner cell layers in spheroids (> 10–45 μm depth) (Fig. 1H), with a higher frequency of type I parasites penetrating to deep layers, compared to type II parasites, and related to total numbers of parasites in spheroids (Fig. 1I). For these lines (type II vs. type I), penetration ratios at 24 h timepoint ranged between 4 and 10% at 20 μm depth to 0.2–1.2% at 30 μm, respectively (Fig. 1F), implying a 2.5–5.5-fold penetration ratio difference between these two strains and similarly rapid decreases in penetration depth ratios for both strains (Fig. 1J). Co-challenge with type I/II tachyzoites corroborated the phenotypical differences between the two parasite lines in spheroids (Fig. S1D, E, F), indicating that soluble factors or putative effects on host cells by either type I/II did not rescue or influence the invasion phenotypes. We conclude that prototypic type I and II *T. gondii* strains colonize spheroids with inherent differences in invasive ability.

### Colonization of spheroids by ***T. gondii*** is rapid, non-disruptive and does not require tachyzoite replication

**Fig. 2:**
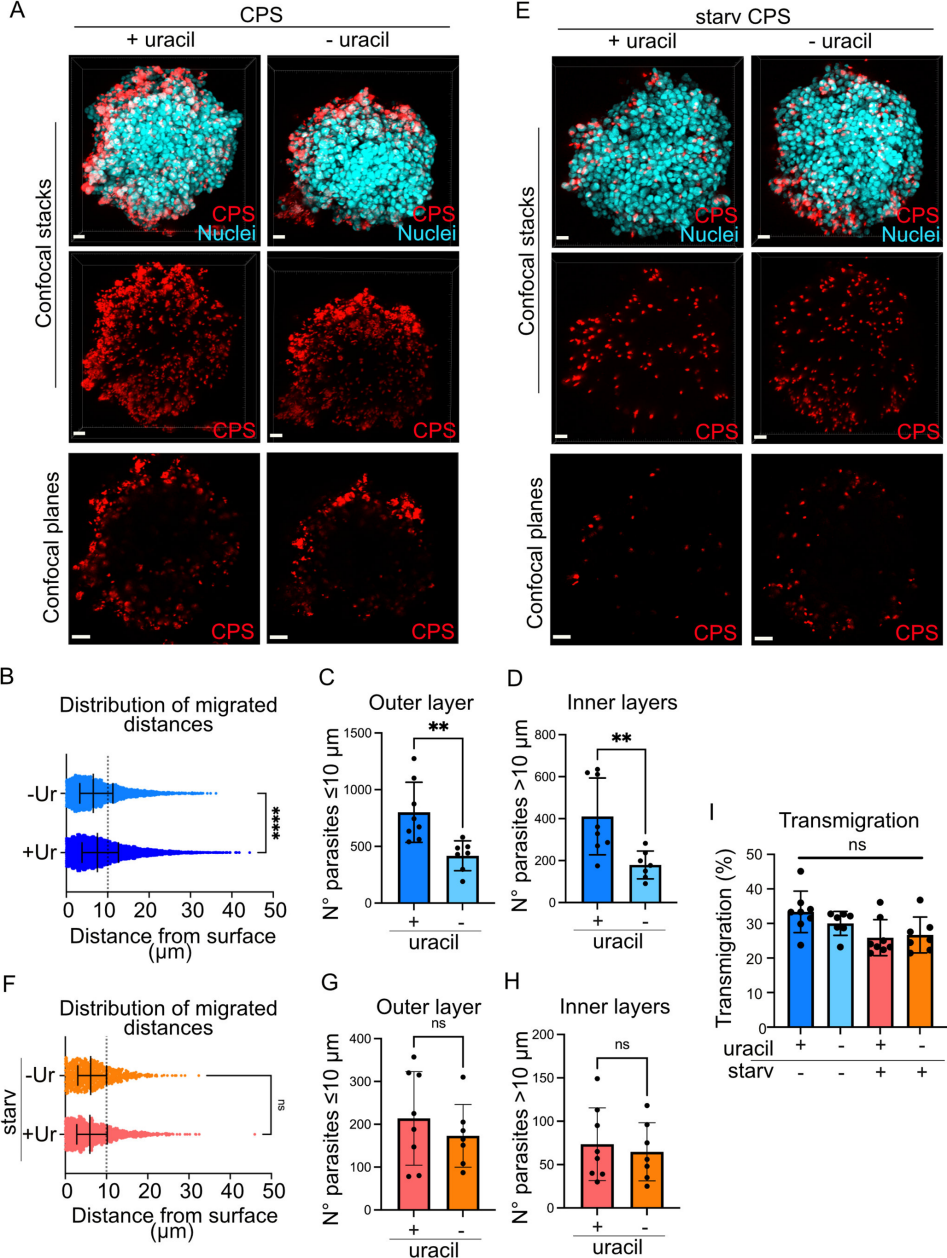
Kinetics of spheroid colonization by *T. gondii* in the absence of parasite replication. **A**. Representative confocal micrographs of bEnd.3 spheroids incubated with freshly egressed mCherry-expressing RH-CPS tachyzoites (5 × 10^4^ cfu *Tg*/spheroid) +/- uracil for 24 h. Confocal stacks and confocal planes (50 μm depth) are shown. Nuclei were stained with DAPI. Scale bars: 20 μm. (**B**,** C**,** D**) Spheroids were incubated with freshly egressed RH-CPS tachyzoites (5 × 10^4^ cfu *Tg*/spheroid) +/- uracil for 24 h (*n* = 4.178–9.789 tachyzoites from 7–8 spheroids). **B**. Graph shows the distribution of migrated distances (µm) by type I non-replicative RH-CPS tachyzoites. Median, 25th and 75th percentiles are indicated. Dotted line indicates depth of 10 μm. **C**,** D**. Bar graphs show the absolute numbers (mean ± SEM) of tachyzoites per spheroid located at ≤ 10 μm (C, outer layer) and > 10 μm (D, inner layers), respectively. **E**. Representative confocal micrographs with conditions identical to (A), except use of uracil-starved (starv, 24 h) RH-CPS tachyzoites. (**F**,** G**,** H**) RH-CPS tachyzoites were uracil-starved for 24 h prior to harvest from host cells before incubation with spheroids (5 × 10^4^ cfu *Tg*/spheroid) +/- uracil for 24 h. (*n* = 833 − 1.640 tachyzoites from 7–8 spheroids) **F**. Graph shows the distribution of migrated distances (µm) by type I non-replicative RH-CPS tachyzoites. Median, 25th and 75th percentiles are indicated. Dotted line indicates depth of 10 μm. **G**,** H**. Bar graphs show the absolute numbers (mean ± SEM) of tachyzoites per spheroid located at ≤ 10 μm (G, outer layer) and > 10 μm (H, inner layers), respectively. **I**. Graph shows the percentage (mean ± SEM) of RH-CPS tachyzoites (from B and F) penetrating > 10 μm related to total numbers of tachyzoites in spheroids (Transmigration) at 24 hpi (*n* = 7–8 spheroids/condition) All data are from 3 independent experiments. (B, F) 2-tailed Mann-Whitney U-test; (**C**, **D**, **G**, **H**) 2-tailed unpaired Student’s t-test; (I) One-way ANOVA followed by Bonferroni’s multiple comparison test. ** *p* < 0.01; **** *p* < 0.0001; ns: non-significant

*T. gondii* tachyzoites are capable of invading the cerebral endothelium in mice. It has been proposed that parasite replication in endothelial cells is required for the parasite to access the brain parenchyma [33]. However, contrasting evidence suggests that tachyzoites can also traverse the BBB without prior replication, via direct transmigration mechanisms [34, 35]. To address if parasite replication was necessary to invade spheroids, we used a uracil-auxotroph *T. gondii* line (CPS, type I), which does not replicate in the absence of added uracil [36]. First, we determined the invasion of spheroids by this line, in presence or absence of uracil (Fig. 2A). Deprivation of uracil, implying abolished parasite replication and likely metabolic stress [36], significantly impacted the distribution of migrated distances (Fig. 2B), with reduced absolute numbers of tachyzoites invading the outer cell layer (≤ 10 μm) (Fig. 2C). Similarly, the absolute numbers of tachyzoites migrating into inner layers decreased (Fig. 2D). Yet, we observed that a number of non-replicating uracil-deprived tachyzoites still reached deeper layers (> 10–40 μm) (Fig. 2B, D). To confirm this observation further, tachyzoites were first deprived of uracil for 24 h in HFF monolayers, harvested and added to spheroids (Fig. 2E). Upon uracil starvation for additional 24 h, tachyzoites were retrieved inside spheroids (up to 40 μm) with non-significant differences in migrated distances between conditions (Fig. 2F), indicating that the phenotype was not rescued by addition of uracil. Lower absolute numbers of tachyzoites were retrieved in spheroids superficially (Fig. 2G, relate to 2 C) and still tachyzoites were located in deeper layers (Fig. 2H, relate to 2D). This evidenced further that a number of tachyzoites reached deep layers of spheroids in absence of replication. Finally, data analyses showed that the ratio (%) of transmigrating parasites (> 10 μm) related to total parasite numbers in spheroids was similar for all conditions (Fig. 2I), corroborating that replication at early or late timepoints was not imperative to reach the inner layers of spheroids.

**Fig. 3:**
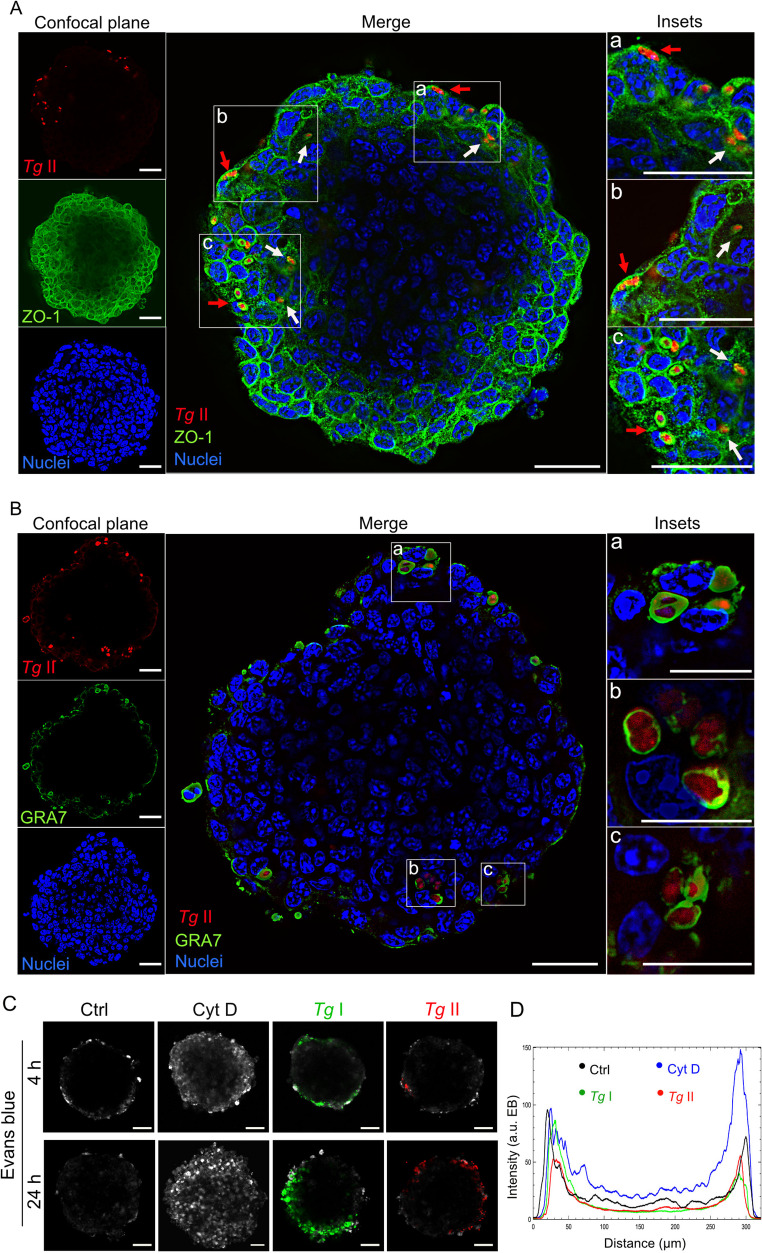
Cellular characterizations of spheroid colonization by ***T. gondii*** tachyzoites **A**. Deconvolved confocal micrographs show localization of type II ME49 tachyzoites (RFP^+^, red), ZO-1 (anti-ZO-1/Alexa 647, green) and nuclei (DAPI^+^, blue) at 50 μm plane depth in spheroids following incubation with 5 × 10^4^ cfu *Tg*/spheroid for 4 h. Insets depict magnifications of merged micrograph. Red- and white-colored arrowheads indicate individual tachyzoites located in the outer cell layer and inner cell layers, respectively. Representative images from multiple experiments, as detailed in Fig. S3. Scale bars: 30 μm, inset scale bars: 15 μm **B.** Deconvolved confocal micrographs show localization of type II ME49 tachyzoites (RFP^+^, red), GRA7 (anti-GRA7/Alexa 647, green) and nuclei (DAPI^+^, blue) at 50 μm plane depth in spheroids following incubation with 5 × 10^4^ cfu *Tg*/spheroid for 16 h. Insets depict magnifications of merged micrograph. Representative images from multiple experiments, as detailed in Fig. S3. Scale bars: 30 μm, inset scale bars: 10 μm **C.** Spheroids were incubated with Evans blue (EB) in control media (Ctrl), cytochalasin D (Cyt D, 20 ng/ml) or challenged with type I (RH-GFP) or type II (ME49-RFP) tachyzoites (2 × 10^5^ cfu *Tg*/spheroid) for 4–24 h. Confocal micrographs show fluorescence signal of EB (grey). Scale bar, 50 μm **D.** Graph shows, for each condition, Evans blue (EB) fluorescence intensity profiles (a.u., arbitrary units) across spheroids, upon treatment as in (C) for 4 h, as detailed in Fig S3F. Representative of 3–4 experiments

Next, to further assess tachyzoite migration prior to replication, we investigated early colonization using wild-type type I and II strains (RH-GFP; ME49-RFP), which typically have a replication time of 6–8 h in vitro [37]. The ability of type I strains to transmigrate across polarized monolayers and the BBB in mice has been documented [19, 34, 35]. In contrast, much less is known about the migratory behavior within tissues of the highly prevalent type II strains in humans [19]. To address this gap, we challenged spheroids with type II ME49-RFP tachyzoites. At 4 h post-challenge, individual tachyzoites were detected within the spheroids, indicating rapid transmigration across the outer cell layer and subsequent passage through TJ (ZO-1)–expressing inner cell layers, in absence of replication (Fig. 3A). Similarly, type I RH-GFP strain also invaded and transmigrated across TJ-expressing cell layers (Fig. S3A), consistent with data at 24 h (Fig. 1D, F). Importantly, at 16 h post-infection, vacuolar structures positive for the marker GRA7 (PVs) and containing replicating type II tachyzoites were detected in both the outer and inner cell layers (Fig. 3B). The latter reveals that *T. gondii* tachyzoites retain the ability to productively invade host cells following transmigration across cell layers, similarly for type I and II (Fig. S3B-E). Because tachyzoite colonization, at these timepoints, occurred without detectable disruption of spheroid gross structural integrity or apparent cell lysis (Fig. 3A; Fig S3C-E), we next assessed transmigration in the presence of the permeability marker EB. Upon challenge with *T. gondii* type I or type II, EB signal intensity was similar to unchallenged condition, contrasting with depolarized, cytochalasin D-treated spheroids (Fig. 3C, D; Fig. S3F-H). This indicates that transmigration occurred without a measurable significant impact on the gross structural integrity of spheroids or measurable sustained effects of depolarization. Together, we conclude that type I and type II *T. gondii* tachyzoites can colonize spheroids by direct transmigration across several TJ-expressing cellular layers in the absence of replication before transmigration.

### ***T. gondii***-infected phagocytes adhere to and colonize spheroids

**Fig. 4:**
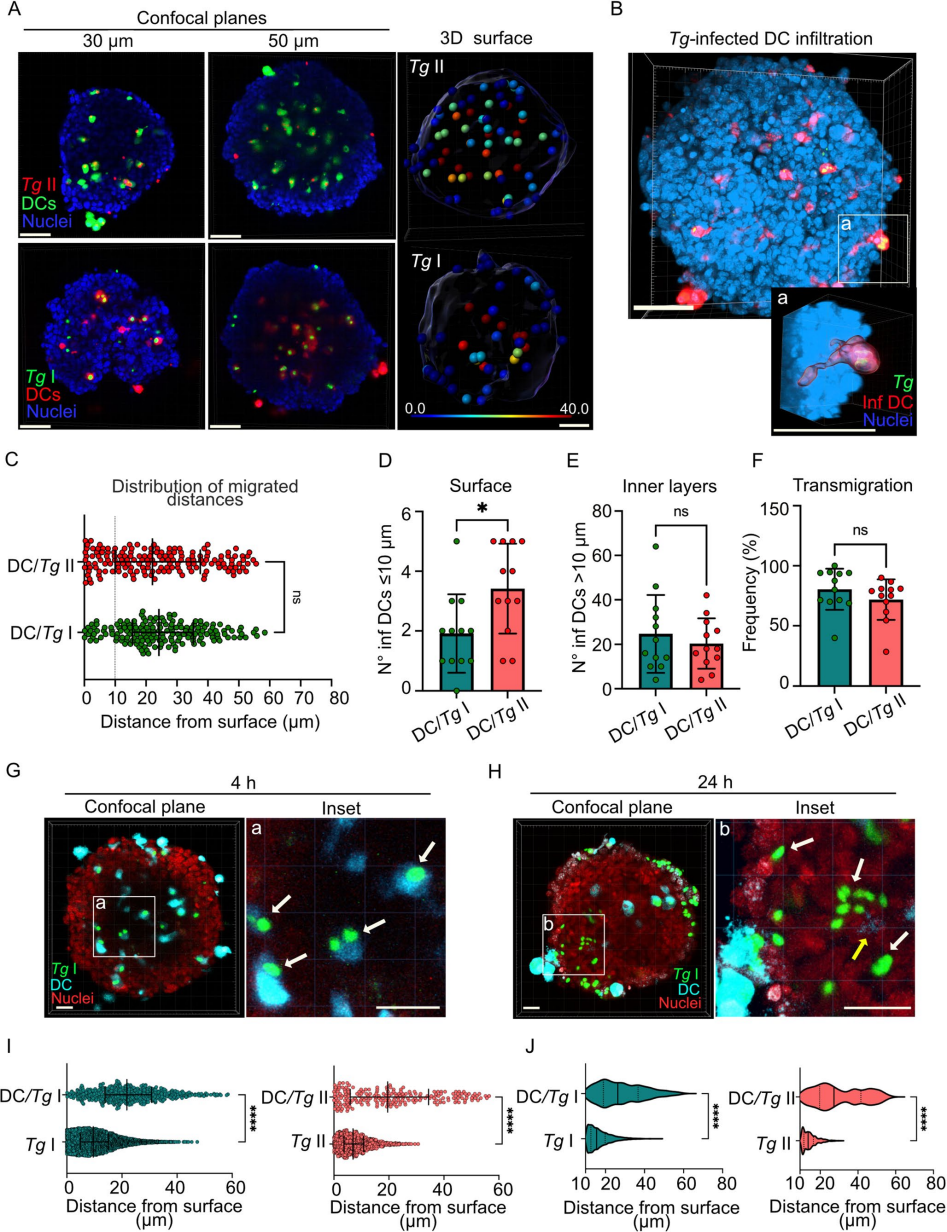
Invasion of spheroids by ***T. gondii***-infected DCs **A**. Confocal micrographs (left panels) show localization of *T. gondii* type I (RH-GFP^+^, green) and II (ME49-RFP^+^, red) *-*infected DCs (CMTMR^+^, red; CFSE^+^, green, respectively) in relation to nuclei (DAPI^+^, blue) at planes of 30 and 50 μm depth inside spheroids. CFSE or CMTMR pre-labelled DCs were challenged with *T. gondii* (RH-GFP, MOI 1; ME49-RFP, MOI 2) to obtain a DC infection frequency of ~ 50% following incubation with spheroids (2 × 10^4^ DCs/~1 × 10^4^ cfu *Tg*/spheroid) for 16 h. 3D surface analysis (right panels) from confocal stacks (0–70 μm depth) show the 3D distribution of type I and II *T. gondii*-infected DCs as indicated under Materials and Methods. The 3D maps show the surface of the spheroids and the infected DCs, represented by dots, color-coded by their distance from the nearest surface (µm). Scale bar: 50 μm **B.** 3D projection and surface analysis micrographs show the localization of RH-GFP-infected DCs (GFP^+^/CMTMR^+^, green/red) on the surface of spheroids at 16 h. Inset shows infiltration of infected DCs toward the inner layer of the spheroids. Scale bars: 50 μm, inset scale bar: 10 μm **C.** Graph shows the distribution of migrated distances by type I and type II infected DCs, respectively, following incubation with 2 × 10^4^ DCs/~1 × 10^4^ cfu *Tg*/spheroid for 16 h. Median, 25th and 75th percentiles are shown. Dotted line indicate depth of 10 μm (*n* = 164 to 182 infected DCs from 12 spheroids/condition) **D**,** E.** Graphs show the absolute numbers (mean ± SEM) of infected DCs per spheroid located ≤ 10 μm from the surface (D) and > 10 μm (E, inner layers) following incubation with 2 × 10^4^ DCs/~1 × 10^4^ cfu *Tg*/spheroid for 16 h. (*n* = 12 spheroids/condition) **F.** Graph shows the percentage (mean ± SEM) of infected DCs penetrating > 10 μm (transmigration) related to total numbers in spheroids, following incubation with 2 × 10^4^ DCs/~1 × 10^4^ cfu Tg/spheroid for 16 h. (*n* = 12 spheroids/condition) **G.** Confocal micrograph shows localization of *T. gondii* type I (RH-GFP^+^, green)*-*infected DCs (CM2FHC^+^, cyan) in relation to nuclei (methyl green^+^, red) at plane of 50 μm depth inside spheroid at 4 h post-challenge. Arrows in inset (a) show association of tachyzoites (GFP^+^) with DCs (CM2FHC^+^). Representative from 3 independent experiments. Scale bar: 20 μm, inset scale bar: 20 μm **H.** Confocal micrograph upon challenge as in (G) but at 24 h timepoint. White arrows in inset (b) show individual and grouped tachyzoites (GFP^+^) and yellow arrow indicates DC membrane remnant (*ghost*, CM2FHC^+^). Scale bar: 20 μm, inset scale bar: 20 μm **I.** Graphs show the distribution of migrated distances by tachyzoites (1 × 10^4^ cfu *Tg*/spheroid) and tachyzoite-infected DCs (2 × 10^4^ DCs/~1 × 10^4^ cfu *Tg*/spheroid) for type I and type II *T. gondii*, respectively, after 16 h. Median, 25th and 75th percentiles are indicated. (*n* = 8.752 tachyzoites (*Tg* I) and 550 infected DCs; 1450 tachyzoites (*Tg* II) and 309 infected DCs, respectively, from 12–17 spheroids/condition) **J.** Violin plots show, for each condition, the distribution of migrated distances by transmigrated (> 10 μm) infected DCs and tachyzoites for type I and II strains, respectively. Median, 25th and 75th percentiles are indicated. (*n* = 4.148 tachyzoites (*Tg* I) and 151 infected DCs; 843 tachyzoites (*Tg* II) and 207 infected DCs, respectively, from 12–17 spheroids/condition) All data are from 3 independent experiments. (C, I, J): 2-tailed Mann-Whitney U-test; (D, E, F): 2-tailed unpaired Student’s t-test. * *p* < 0.05; **** *p* < 0.0001; ns: non-significant

Parasitized phagocytes facilitate the dissemination of *T. gondii*. Differences in systemic spread and cerebral capillary sequestration of *T. gondii*-infected DCs have been associated with the parasite’s archetypal lineages [22, 35]. However, whether parasite lineage impacts transmigration across polarized endothelium and migration in tissue-like structures has remained unexplored. To this end, we first challenged spheroids with DCs infected with *T. gondii*. Interestingly, penetration of the spheroid was observed by *T. gondii*-infected DCs, in a similar fashion for type II (ME49)- and type I (RH)-infected DCs (Fig. 4A, B, Movie S4), rapidly reaching considerable penetration depths up to ~ 60 μm (Fig. 4C, Movie S5). Further, type II-infected DCs exhibited a superior absolute number of infected DCs adhering to the surface of organoids (Fig. 4D), with comparable relative numbers of infected DCs migrating into the spheroids (Fig. 4E) and similar transmigration frequency (Fig. 4F). By 4 h post-inoculation, *T. gondii* signal was associated with pre-labeled DCs, indicating transportation by infected DCs (Fig. 4G). In contrast, after 24 h, individual or groups of tachyzoites without association to DC dye and pre-labeled DC membrane remnants (*ghosts*) were detected, indicating egress from infected DCs and further spread (Fig. 4H). Jointly, this suggests that DC-mediated transport of tachyzoites to deeper cellular layers facilitates rapid colonization of areas that free tachyzoites cannot initially access.

Next, given that other mononuclear phagocytes, such as macrophages, also contribute to *T. gondii* dissemination [30, 38], we challenged spheroids with *T. gondii*-infected macrophages, differentiated using M-CSF or GM-CSF. Both macrophage types infiltrated the spheroids, showing comparable or greater attachment to the spheroid surface but lower transmigration frequencies than DCs (**Fig. ****S4****A-D** and Fig. 4D, F). These findings align with their known migratory profiles observed in 2D assays and in vivo models [30, 38, 39]. Finally, in parallel experiments, we compared the two modes of colonization -free tachyzoites and infected DCs- for type I and II *T. gondii*. For both lines, *T. gondii*-infected DCs consistently reached longer distances compared with free tachyzoites (Fig. 4I). Further comparisons between subpopulations of infected DCs and tachyzoites that had penetrated spheroids (> 10 μm), corroborated these differences (Fig. 4J), indicating they did not depend primarily on the initial attachment frequency to the spheroid surface (Fig. 4D). Jointly, the data show colonization of spheroids by both type I- and type II-infected DCs, with a superior surface adhesion to spheroids by type II (ME49)-infected DCs. This motivated a further analysis of the interactions of infected DCs with spheroids.

### Characterization of infected DC migration into spheroids

**Fig. 5:**
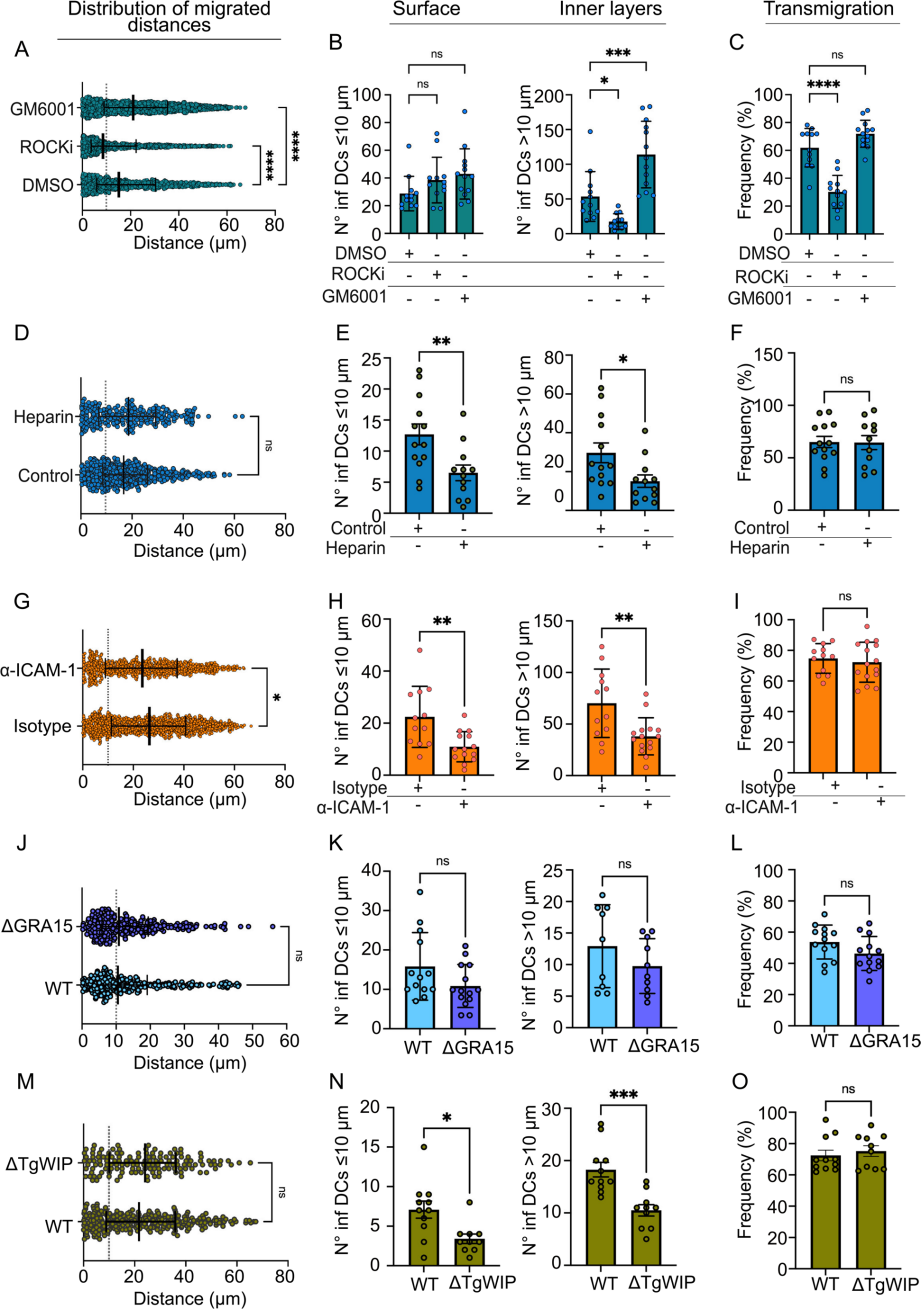
Determinants and migration modes of *T. gondii*-infected DCs into spheroids (**A** – **C**). CMF2HC pre-labelled DCs were challenged with *T. gondii* (type II, PRU-GFP, MOI 2, 4 h) to obtain a DC infection frequency of ~ 50% followed by incubation with inhibitors GM6001, ROCKi or mock-treated with DMSO (control) for 1 h. Spheroids were challenged with treated cells suspensions (2 × 10^4^ DCs/1 × 10^4^ cfu *Tg/*spheroid) for 16 h **A**. Distribution of migrated distances (µm) by infected DCs in spheroids. Median, 25th and 75th percentiles are shown. Dotted line indicate depth of 10 μm (*n* = 1.030–2.013 infected DCs) **B**. Graphs show the absolute numbers (mean ± SEM) of infected DCs per spheroid at ≤10 μm (surface, left) and > 10 μm (inner layers, right) (*n* = 12–13 spheroids/condition) **C**. Graphs shows the percentage (mean ± SEM) of infected DCs per spheroid penetrating > 10 μm related to the total number of infected DCs in the spheroid, defined as transmigration (*n* = 12–13 spheroids/condition) (**D – F**). CMF2HC pre-labelled DCs were challenged with *T. gondii* (Type II, PRU-GFP, MOI 2, 4 h) to obtain a DC infection frequency of ~ 50%. Immediately after addition of heparin (500 U/ml) or control media, cell suspensions were transferred to spheroids and incubated for 16 h **D**. Distribution of migrated distances (µm) by infected DCs in spheroids. Median, 25th and 75th percentiles are shown. Dotted line indicate depth of 10 μm (*n* = 355–510 infected DCs) **E**. absolute numbers (mean ± SEM) of infected DCs per spheroid at ≤ 10 μm (surface, left) and > 10 μm (inner layers, right) (*n* = 12–13 spheroids/condition) **F**. Percentage (mean ± SEM) of infected DCs per spheroid penetrating > 10 μm, defined as transmigration (*n* = 12–13 spheroids/condition) (**G – I**). CMF2HC pre-labelled DCs were challenged with *T. gondii* (type II, PRU-GFP, MOI 2, 4 h) to obtain a DC infection frequency of ~ 50% followed by incubation with anti-ICAM-1 antibody or isotype for 1 h. Spheroids were challenged with treated cells suspensions (2 × 10^4^ DCs/1 × 10^4^ cfu *Tg/*spheroid) for 16 h **G**. Distribution of migrated distances (µm) by infected DCs in spheroids. Median, 25th and 75th percentiles are shown. Dotted line indicate depth of 10 μm (*n* = 1.033–1.339 infected DCs) **H**. Absolute numbers (mean ± SEM) of infected DCs per spheroid at ≤ 10 μm (surface, left) and > 10 μm (inner layers, right) (*n* = 12–14 spheroids/condition) **I**. Percentage (mean ± SEM) of infected DCs per spheroid penetrating > 10 μm, defined as transmigration (*n* = 12–15 spheroids/condition) (**J – L**). CMTMR pre-labelled DCs were challenged with GFP-expressing wild type (*WT*) or *ΔGRA15* tachyzoites (type II, MOI 2) to obtain a DC infection frequency of ~ 50% followed by incubation with spheroids (2 × 10^4^ DCs/1 × 10^4^ cfu *Tg*/spheroid) for 16 h **J.** Distribution of migrated distances (µm) by infected DCs in spheroids. Median, 25th and 75th percentiles are shown. Dotted line indicate depth of 10 μm (*n* = 610–697 infected DCs) **K**. Absolute numbers (mean ± SEM) of infected DCs per spheroid at ≤ 10 μm (surface, left) and > 10 μm (inner layers, right) (*n* = 9–14 spheroids/condition) **L**. Percentage (mean ± SEM) of infected DCs per spheroid penetrating > 10 μm, defined as transmigration (*n* = 13 spheroids/condition) (**M – O**). CMF2HC pre-labelled DCs were challenged with GFP-expressing wild type (*WT*) or *ΔTgWIP* tachyzoites (type I, MOI 1, 4 h) to obtain a DC infection frequency of ~ 50% followed by incubation with spheroids (2 × 10^4^ DCs/1 × 10^4^ cfu *Tg*/spheroid) for 4 h **M**. Distribution of migrated distances (µm) by infected DCs in spheroids. Median, 25th and 75th percentiles are shown. Dotted line indicate depth of 10 μm (*n* = 139–279 infected DCs) **N**. Absolute numbers (mean ± SEM) of infected DCs per spheroid at ≤ 10 μm (surface, left) and > 10 μm (inner layers, right) (*n* = 10–11 spheroids/condition) **O**. Percentage (mean ± SEM) of infected DCs per spheroid penetrating > 10 μm, defined as transmigration (*n* = 10–11 spheroids/condition) All data are from 9–15 spheroids per condition from 3 independent experiments. (A, D, G, J, M): 2-tailed Mann-Whitney U-test; (B, C): One-way ANOVA followed by Bonferroni’s multiple comparison test. (E, F, H, I, K, L, N, O): 2-tailed unpaired Student’s t-test. * *p* < 0.05; ** *p* < 0.01; *** *p* < 0.001; **** *p* < 0.0001; ns: non-significant

We previously reported induction of amoeboid-like mode of motility by *T. gondii* in infected DCs in extracellular matrix constructs [40] and infection-dependent adhesion to endothelial monolayers [41]. However, the mode of migration of parasitized DCs within the multicellular layers of spheroids, that better resemble tissue structures, has remained unknown. To address this, we pre-treated infected DCs with ROCK inhibitor (ROCKi), know to inhibit adhesion-independent amoeboid motility [42], or with the metalloproteinase inhibitor GM6001, known to inhibit adhesion-dependent interstitial motility [43]. Interestingly, ROCKi reduced migrated distances while GM6001 elevated migrated distances (Fig. 5A). Detailed analysis revealed that the treatment did not significantly affect numbers of infected DCs at the surface of the spheroid (Fig. 5B), indicating maintained adhesion and invasion of the outer cell layer. In sharp contrast, treatments had profound effects on numbers of infected DCs in deeper layers (> 10 μm), ROCKi reducing and GM6001 elevating numbers (Fig. 5B). Similarly, effects on transmigration were also demonstrated when relating to total numbers of infected DCs in spheroids (Fig. 5C). These data indicated that *T. gondii*-infected DCs primarily use amoeboid motility to migrate into spheroids and that inhibition of adhesion-dependent interstitial motility elevate their migration into spheroids. The impact of the initial adhesion of infected DCs to the surface of spheroids was confirmed by heparin treatment, which reduced adhesion to the surface and also migration inside spheroids (Fig. 5D, E), but not transmigration frequency per se (Fig. 5F).

Next, because *T. gondii* effectors secreted into the host cell and host cell adhesion molecules modulate the migratory features of parasitized leukocytes in vitro [30, 41], we hypothesized that similar effects could be in play in spheroids. Specifically, because GRA15 impacts ICAM-1 expression [41] and both molecules are implicated in the sequestration of infected DCs in the cerebral microvasculature [35], we tested the effects of ICAM-1 blockade. Inhibition of ICAM-1 reduced migrated distances (Fig. 5G), numbers of infected DCs in outer layer and deeper layers (Fig. 5H), but not transmigration frequency per se (Fig. 5I). Finally, we assessed the impact of GRA15 on the migration of infected DCs. DCs infected with wild-type or *ΔGRA15* mutants migrated similarly in the assays (Fig. 5J-L). This indicates that GRA15 primarily impacts the adhesion of infected DCs in vitro and in vivo [35], rather than their migratory properties. In contrast, deletion of the effector TgWIP, known to be implicated in DC migratory activation [29] and sequestration in cerebral capillaries [35], markedly reduced the number of infected DCs entering the spheroids but did not affect their maximal migration distances or transmigration frequency in relation to total numbers in spheroids (Fig. 5M-O). These findings suggest potential effects at multiple levels, such as surface adhesion and entry mechanisms into spheroids. Together, the data provide proof-of-concept that secreted *T. gondii* effectors modulate spheroid colonization by infected DCs. Moreover, the results indicate that ICAM-1 facilitates the adhesion of infected DCs to the endothelial surface and that these cells primarily migrate within spheroids through an amoeboid mode of motility.

#### Colonization of organoids derived from cortical tissue by tachyzoites and infected DCs

**Fig. 6:**
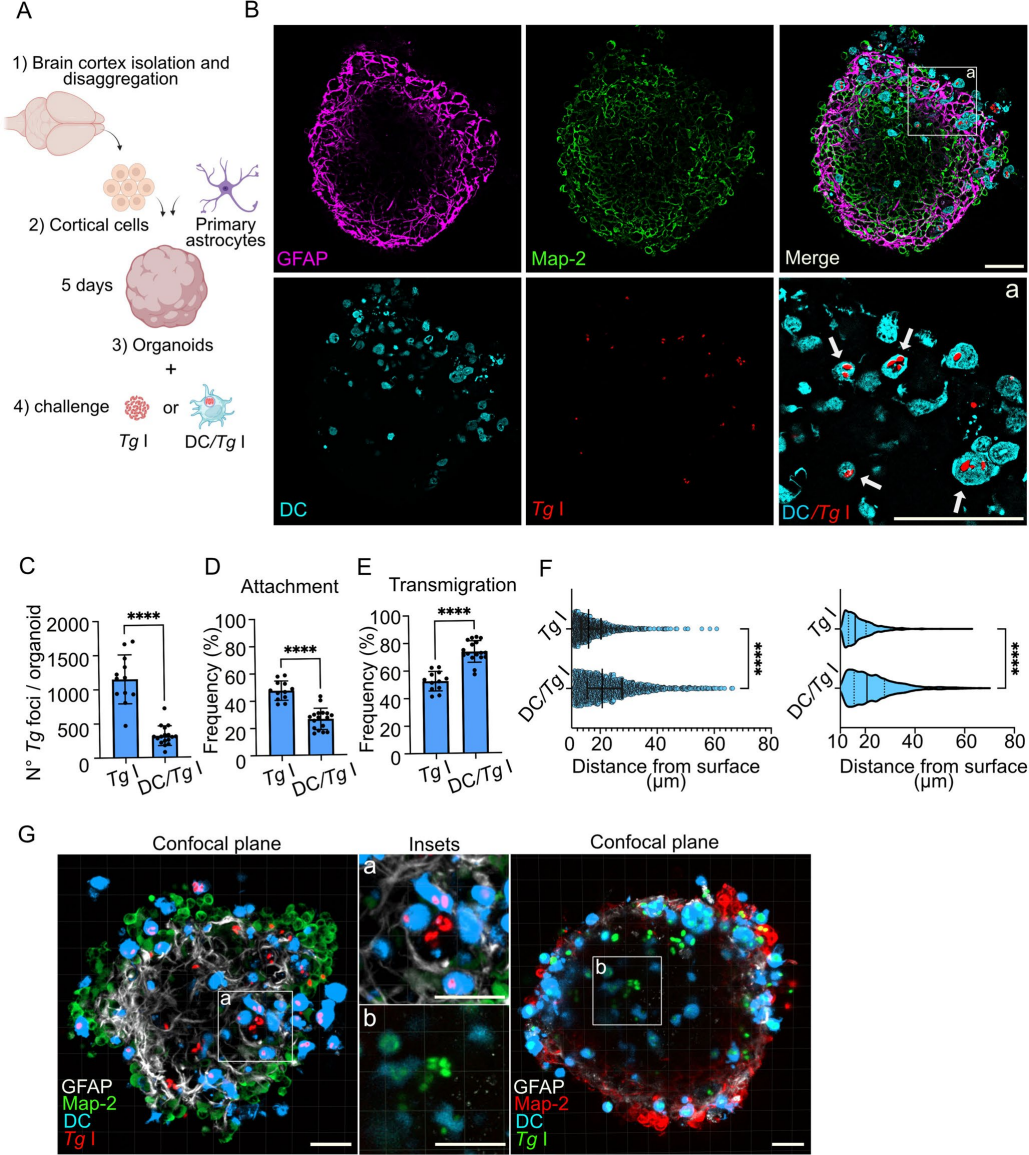
Penetration of brain organoids by parasitized DCs and by tachyzoites **A**. Cartoon shows primary cortical organoids set up. Brain cortex cell suspensions were mixed at 1:1 ratio with primary astrocytes. Cell mix (5 × 10^3^/well) was seeded into U-bottom non-adherent plates and incubated for 5–6 days. Organoids (1/well) were incubated with free tachyzoites or tachyzoite-infected DCs, fixed and stained with indicated antibodies **B**. Confocal micrographs show localization of *T. gondii* type I (RH-mCherry^+^, red)*-*infected DCs (CM2FHC^+^, cyan) in relation to astrocytes (GFAP^+^, magenta) and neurons (Map-2^+^, green) at 50 μm depth inside organoids. CM2FHC pre-labelled DCs were challenged with type I RH-mCherry tachyzoites (MOI 1) to obtain a DC infection frequency of ~ 50% followed by incubation with organoids (2 × 10^4^ DCs/1 × 10^4^ cfu *Tg*/organoid) for 16 h. Organoids were then fixed and stained with indicated antibodies. Inset (a) shows magnification of *T. gondii* (red)- infected DCs (cyan) inside organoid, indicated by white arrows. Scale bars, 50 μm **C**. Graphs show the absolute numbers (mean ± SEM) of type I RH free-tachyzoites (*Tg*) compared to type I RH-infected DCs (DC/*Tg* I) per organoid, following incubation with 1 × 10^4^ cfu *Tg* or DC/*Tg* I per organoid for 16 h (*n* = 12 (*Tg* I) and 17 (DC/*Tg* I) spheroids) **D**,** E**. Graphs show the absolute numbers (mean ± SEM) of tachyzoite (*Tg*) or infected DCs (DC/*Tg* I) per organoid at ≤ 10 μm (surface, D) and > 10 μm (deeper layers, E), following inoculation with 1 × 10^4^ cfu *Tg* or DC/*Tg* I per organoid for 16 h. (*n* = 12 (*Tg* I) and 17 (DC/*Tg* I) spheroids) **F**. Graph shows the distance to the nearest organoid surface (µm) of type I RH free-tachyzoites (*Tg*) compared to RH-infected DCs (DC/*Tg* I) following incubation with 1 × 10^4^ cfu *Tg* or DC/*Tg* I per organoid for 16 h. Median, 25th and 75th percentiles are shown (*n* = 13.873 tachyzoites and 5.300 infected DCs from 12–17 spheroids/condition) **G**. Representative confocal micrographs at 50 μm depth plane show groupings of non-DC associated *T. gondii* tachyzoites in organoids at 20–24 h timepoints. Insets (a, b) indicate, respectively, *T. gondii* (RH) tachyzoites (mCherry^+^, red or GFP^+^, green) in the absence of associated DC labeling (CM2FHC^+^, cyan). Scale bars, 30 μm All data are from 3 independent experiments. (C, D, E): 2-tailed unpaired Student’s t-test; (F): 2-tailed Mann-Whitney U-test; **** *p* < 0.0001

After crossing endothelium, *T. gondii* tachyzoites or *T. gondii*-infected leukocytes enter deeper layers in the brain parenchyma, encountering neurons, astrocytes and glia cells [34]. To address migration at this phase of the infection, we constructed organoids derived from cells suspensions purified from murine cortices (Fig. 6A). Cells expressing the neuronal marker MAP2 and astrocyte marker GFAP formed an organized network with a more peripheral layer of GFAP-expressing cells (Fig. 6B). Next, organoids were challenged with free tachyzoites or with tachyzoite-infected DCs for 16 h (Fig. 6B). For comparisons with infected DCs, type I tachyzoites were chosen because of their superior migratory capability compared to type II (Fig. 1 and S3). Interestingly, higher total numbers of tachyzoites invaded organoids when related to *T. gondii*-infected DCs (Fig. 6C) or compared with invasion of spheroids (Fig. 1F). Detailed analysis unveiled a superior relative localization of tachyzoites to the outer layer of organoids (≤ 10 μm) (Fig. 6D), while *T. gondii*-infected DCs exhibited superior transmigration frequency (Fig. 6E) and, importantly, a deeper penetration of the organoids (Fig. 6F). Passed 20 h infection of DCs, groupings of tachyzoites in absence of DC dye started to appear (Fig. 6G), indicating egress from DCs and mirroring the patterns of post-replicative spread observed in spheroids (Fig. 4). Finally, attempts to generate combined constructs by mixing cell suspensions of bEnd.3 endothelial cells, purified cortical cells and primary astrocytes yielded juxtaposed constructs of cortical organoids and bEnd.3 spheroids (Movie S6) and represents a challenge for future research. We conclude that both tachyzoites and *T. gondii*-infected DCs invade and colonize cortical-tissue organoids, with a superior migration of *T. gondii*-infected DCs compared to free tachyzoites.

## Discussion

Our findings show that brain endothelial cell (b.End3) spheroids offer a practical and reproducible model for investigating *T. gondii* translocation across the BBB. Prior spheroid and organoid studies have provided important insights, including reports of *T. gondii* cyst formation in neurospheres [44], infection of placental trophoblast spheroids [45], and parasite growth in stem cell-derived cerebral organoids [46]. In contrast, our work specifically leverages spheroid and organoid systems to model BBB traversal and brain colonization. To achieve this, we prioritized controlling the robust expression of TJ markers and monitored barrier impermeability during the assays. Instability of TJ marker expression during extended culture in 2D systems using primary endothelial cells or cell lines is a well-recognized limitation [47]. Notably, spheroid colonization occurred without detectable loss of barrier integrity or evidence of cell lysis, offering insights into the non-lytic mechanisms observed around cerebral capillaries in vivo [33–35]. In particular, only sparse, localized increases in EB permeability were detected adjacent to certain replicative parasite foci in murine cortex [34]. These findings align with previous results from transwell models using polarized epithelial and brain endothelial cell monolayers, which exhibited only modest changes in transcellular electrical resistance (TCER) during transmigration, while maintaining impermeability to dextran-FITC and showing no signs of cellular lysis [17, 18]. To our knowledge, this is the first demonstration of such a phenomenon in a 3D culture system that more closely mimics the cellular architecture of in vivo tissues. Collectively, data from spheroids, polarized monolayers, and mouse models indicate that early colonization by *T. gondii* occurs without widespread barrier disruption or microhemorrhage, but rather through sparse, focal, and likely transient destabilization of the cellular barrier. Furthermore, invasion of the CNS by *T. gondii* is a relatively rare event, related to numbers of invasion events in other peripheral organs [34]. The spheroid model enabled reproducible acquisition of both quantitative and qualitative data under controlled conditions, thereby extending and corroborating in vivo observations that are otherwise difficult to measure [34, 35].

A limitation of this model system, however, is the absence of immune cells and a circulatory network, factors that naturally modulate the dynamics of inflammation, cellular adhesion and migration. Although freshly isolated primary brain endothelial cells possess certain phenotypic advantages over cell lines [47], their substantial heterogeneity [48], limited yield following purification and characterization [17], slow growth and progressive loss of TJ marker expression upon passaging [8, 49] made them unsuitable for our purposes. Nonetheless, our findings provide proof-of-concept that compact, TJ-expressing bEnd.3 spheroids constitute a robust and easily scalable platform (96-well plate, 72 h culture) to investigate the molecular and cellular determinants of *T. gondii* tissue colonization. This model also holds potential for screening parasite mutant phenotypes and pharmacological compounds; tasks that are otherwise labor-intensive, time-consuming, and challenging to assess in vivo in mice. Finally, this system can be readily adapted to investigate host-pathogen interactions of other microbes.

The data establish that free (extracellular) *T. gondii* type I and II tachyzoites are capable of migrating across several layers of TJ-expressing cells and subsequently invading cells within the deeper regions of spheroids. Their characteristic gliding motility [19], likely facilitated this traversal paracellularly [18]. Remarkably, tachyzoites reached depths of 30–40 μm, corresponding to passage through 3–5 layers of TJ-expressing cells, a phenomenon not previously documented under controlled conditions. Moreover, type I tachyzoites (RH) exhibited a clear advantage over type II strains (ME49, PRU) in both migration frequency and maximum depth achieved. These findings extend prior studies based on polarized endothelial and epithelial monolayers [18, 19, 50] and are consistent with observations in mouse brain, where quantifications remain challenging [35]. Specifically, the enhanced migratory ability of type I (RH) tachyzoites is likely attributable to their superior gliding motility, as demonstrated in 2D systems, and to their higher transmigratory capability across polarized monolayers [19]. The cellular wall of the human or murine BBB is approximately 10 μm thick [7], yet tachyzoites in our spheroid model migrated up to 50 μm, far exceeding the physiological barrier thickness. In our experience, *T. gondii* tachyzoites exhibit their most intense gliding motility and invasive capacity immediately following egress from host cells, *strongly* emphasizing the importance of using freshly egressed tachyzoites in experimental settings. Moreover, both gliding motility and invasiveness decline over time, in a strain-dependent manner [19, 34, 35].

We demonstrate that parasite replication is not required for transmigration across layers of TJ-expressing endothelial cells, using non-replicative uracil-auxotrophic parasites (CPS). Remarkably, a subset of these parasites was able to migrate deep into the spheroids (> 30 μm) even after 24–48 h of uracil starvation. Furthermore, following traversal of cellular barriers, we show that tachyzoites are able to resume replication. Thus, spheroid models enabled quantitative analyses of endothelial cell invasion, specifically targeting the outer cell layer (≤ 10 μm), in agreement with in vivo findings in brain endothelium [33–35]. Moreover, the spheroids facilitated simultaneous measurements of direct transmigration by tachyzoites across TJ-expressing endothelial layers, independent of parasite replication. These findings align with reports of rapid, direct tachyzoite penetration from cerebral microvessels into the brain parenchyma [34, 35]. Taken together, we postulate that these specific invasive properties equip *T. gondii* with an efficient mechanism for tissue dissemination, particularly for crossing the BBB, but likely also for traversing other biological barriers, as previously suggested in ex vivo intestinal models [19]. The spheroid and organoid cell constructs enabled both qualitative and quantitative assessment of tachyzoite tissue invasion over time (4, 16, 24 h) in a controlled manner, overcoming certain limitations of current in vivo imaging approaches. While tachyzoites readily invaded both types of constructs, we noted a preferential invasion of organoids. Hypothetically, this may reflect the restrictiveness provided by TJ-expression in endothelial spheroids, while astrocytes and neurons lack TJs and form a more permissive barrier. Jointly, we propose that the present findings likely recapitulate processes occurring at the BBB in vivo.

Our findings demonstrate that parasitized phagocytes can penetrate spheroids and organoids, transporting tachyzoites across multiple layers of TJ-expressing endothelial cells or primary cells expressing astrocytic and neuronal markers. Specifically, infected DCs and macrophages migrated deeper within spheroids and organoids than free tachyzoites, rapidly reaching distances up to 60–70 μm, corresponding to 5–8 cellular layers. In contrast to the superior migration of type I (RH) tachyzoites, both type I- and type II-infected DCs migrated similarly in spheroids. Notably, type II-infected DCs exhibited greater adhesion to spheroid surfaces, a phenomenon implicating ICAM-1 and consistent with the ICAM-1-mediated elevated sequestration of type II-infected DCs in cerebral microvasculature [35]. Together, these observations indicate that although type I tachyzoites exhibit greater transmigration capacity [19, 50], type II parasites may depend more on phagocyte-mediated transport, consistent with findings of systemic dissemination after adoptive transfer of infected DCs in mice [22]. Moreover, while some infected DCs adhered to the spheroid surface, others invaded and migrated within the spheroids. The role of leukocyte-mediated transport, including delivery to CNS vasculature, has been extensively studied [21, 51]. Recent findings, however, indicate that persistent sequestration of infected DCs in cortical capillaries is more common than direct transmigration, although transmigration does occur, particularly under inflammatory conditions [35]. These observations suggest that both pathways likely operate in vivo, and future studies using spheroids and organoids should aim to identify the cues that determine whether infected phagocytes adhere (i.e., sequester in vivo) or transmigrate. One possibility is that infected DCs preferentially sequester early during infection and shift toward transmigration later in response to inflammatory and chemokine cues from the brain parenchyma. While this hypothesis warrants further investigation, recapitulating such inflammatory and chemotactic signals in spheroids remains challenging. Nevertheless, we provide evidence that blockade of DC adhesion using heparin, and specifically targeting ICAM-1 by antibody blockade, reduces the number of infiltrating DCs. Because GRA15 upregulates ICAM-1 [41], likely via NF-κB activation [52], and endothelial NF-κB signaling (through TLR/Myd88) mediates neuronal responses to inflammation [53], it is plausible that GRA15-mediated NF-κB activation contributes to CNS colonization. However, DCs infected with a GRA15-deficient mutant exhibited similar migration patterns to wild-type inside spheroids, suggesting that GRA15 primarily impacts initial adhesion to endothelium [35], likely via ICAM-1, rather than influencing amoeboid migration per se. This aligns with the greater in vivo sequestration observed for type II strains (PRU, ME49) compared to type I (RH), highlighting the significant roles of the effectors GRA15 and TgWIP [35]. In stark contrast, deletion of TgWIP substantially decreased the numbers of infected DCs entering the spheroids. This striking phenotype likely results from cytoskeletal perturbations [29] affecting several key stages of DC infiltration, such as initial adhesion, translocation, and intratissue amoeboid migration, and therefore merits further investigation. Additionally, recent findings that *T. gondii* promotes CCR7-mediated chemotaxis also in macrophages and monocytes through the co-operative action of various effectors [30, 38], raise interesting possibilities. Given that the BBB endothelium constitutively expresses CCL19 [54], CCR7 has been proposed to facilitate the migration of circulating CCR7-expressing T cells across the BBB [55]. However, direct evidence for a role of endothelial CCL19 in facilitating immune cell trafficking to the CNS in non-inflammatory contexts remains absent and merits further investigation.

The data further reinforce and extend to TJ-expressing 3D endothelial constructs, the previously described motility mode switches in infected DCs, transitioning between adhesion-dependent and amoeboid migration on monolayers and within matrices [25, 40, 56, 57]. Collectively, the findings indicate a bias toward amoeboid motility in infected phagocytes within spheroids. Finally, the spatio-temporal dynamics of spheroid and organoid colonization represent key areas for future research, with the potential to sharpen our comprehension of CNS colonization processes. For parasitized phagocytes, it is particularly important to identify the factors that determine adhesion (i.e., sequestration in the microvasculature in vivo) versus transmigration. Likewise, the lineage-related mechanisms that influence whether extracellular tachyzoites invade endothelial cells or transmigrate into the parenchyma remain to be clarified.

Altogether, we propose that the diverse yet covert colonization strategies demonstrated in spheroids and organoids, namely active invasion by extracellular tachyzoites and transport via phagocytes, may provide *T. gondii* with complementary advantages for establishing latent CNS infection. These rapid, non-disruptive processes likely reduce host-damaging inflammation while ensuring efficient access to the parenchyma for conversion to chronic stages. Consistent with this, primary CNS infection with subsequent establishment of latency can remain asymptomatic in humans [14]. From an evolutionary standpoint, such discreet colonization may enhance parasite persistence and therefore promote transmission to the feline definitive host through predation.

## Materials and methods

### Mice

All experiments were performed using male and female C57BL/6NCrl mice pups (strain code 027, Charles River), aged 1–3 days. Dams were housed in a ventilated facility, provided with unrestricted access to tap water and food, and kept under a 12-hour light/dark cycle at a temperature of 20–22 °C.

### Parasite culture and cell lines

*T. gondii* tachyzoites of type I: RH-GFP [58], RH CPS-mCherry [36], RH*ΔTgWIP*-GFP [29]; type II: ME49-RFP [59], PRU-GFP [52] and PRU*ΔTgGRA15*-GFP [52], were maintained by serial 48 h passaging in human foreskin fibroblasts, HFFs (ATCC, CRL-2088). HFFs and murine brain endothelial cells, bEnd.3 (ATCC, CRL-2299) [32], were cultured in High glucose Dulbecco’s modified Eagle’s medium (DMEM, VWR) supplemented with 10% heat inactivated fetal bovine serum (FBS, HyClon), 20 µg/ml gentamicin (Gibco) and 10 mM HEPES (HyClone). Cells and parasites were cultured at 37 °C, 5% CO_2_ in a humidified atmosphere. All cultures were regularly tested for *Mycoplasma*.

### bEnd.3 spheroids

Spheroids were generated as previously described for other cell lines [31]. Briefly, U - bottom 96-well plates were coated with sterile 1% (w/v) ultrapure agarose (Invitrogen), dissolved in ultra-pure milli-Q water. bEnd.3 cells were trypsinized (trypsin/EDTA, Thermo Scientific), resuspended in DMEM with 10% FBS (5 × 10^4^ cell/ml), seeded into the pre-coated plates (5 × 10^3^ cell/well) and incubated at 37 °C with 5% CO2 for 72 h to allow the assembly of spheroids. bEnd.3 cells consistently formed solid spheroidal structures. Typically, a single spheroid of ~ 400 μm diameter was obtained per well.

### Polymerase chain reaction (PCR)

For bEnd.3 monolayers or spheroids, total RNA was extracted using RNeasy mini kit (Qiagen) according manufacture’s protocol. The RNA was quantified using a NanoDrop 1000. cDNA was synthesized with Maxima first strand cDNA synthesis kit (ThermoFisher). Real-time PCR was performed using 50 ng cDNA, 200 nM forward and reverse primers (Table S1) and SYBR green PCR master kit (Kapa Biosystem). GAPDH was used as house-keeping gene to calculate relative expression (2^−∆∆CT^).

### Permeability assay

bEnd.3 spheroids were washed with DMEM 10% FBS and incubated with DMEM 10% FBS, 0.375 mg/ml Evans blue (EB, Sigma-Aldrich), 5% bovine serum albumin (BSA, Sigma-Aldrich) +/- 20 ng/ml cytochalasin D (Sigma-Aldrich) at 37 °C, 5% CO2 for indicated time. When indicated, spheroids were challenged with *T. gondii* tachyzoites, as described below, prior to permeability assay. Then, samples were washed 3 times and mounted in PBS (Gibco) for imaging with a Zeiss LSM 800 confocal microscope. Confocal micrographs at 50 μm depth were collected using a 20x objective. Images were analyzed with Fiji/ImageJ software. A rectangular region of interest (ROI) was created in the midline of each spheroid and fluorescence intensity profiles of EB were generated with the *Plot Profile* tool. Data tables were exported and analyzed with GraphPad Prism v.10 software.

### Organoids

Primary astrocytes were generated as previously described [60]. Brain cortices from 1–3-day-old mice were mince in ice-cold Ca^2+/^Mg^2+^ free Hank’s buffered salt solution (HBSS, Gibco). Tissue was centrifuged at 1000 rpm for 10 min at 4 °C and incubated with 0.125% trypsin (Sigma) in HBSS for 15 min at RT. Tissue was carefully resuspended in 6 ml DMEM F-12 (Gibco) supplemented with 10% FBS, 2mM L-glutamine and 10 ug/ml gentamicin, centrifuged at 1000 rpm for 10 min, and transferred into T-75 culture flask in 12 ml DMEM F-12 supplemented with 10% FBS, 20 μm/ml gentamicin and 1X G5 supplement (ThermoFisher). Cells were incubated at 37 °C with 5% CO2 for 2–3 weeks.

Brain cortical cells were isolated from 1 to 3 day old male and female C57BL/6NCrl mice. Cortices were minced to 1 mm pieces, digested with 0.25% trypsin (Sigma) at 37 °C for 10 min and carefully resuspended. After settling (2 min), the supernatant containing cortical cells was collected, pelleted at 200xg for 4 min and resuspended in Neurobasal media (Thermofischer) supplemented with B27 (ThermoFischer), 0.5 mM L-glutamine and 20 µg/ml gentamycin. Astrocytes and cortical cells (2.5 × 10^3^ each/well) were seeded into pre-coated plates in complete neuronal medium and incubated at 37 °C with 5% CO2 for 5–6 days to allow the assembly of cortical organoids. One organoid with a diameter of ~ 400–500 μm was obtained per well.

### Infection of bEnd.3 spheroids and organoids

BBB spheroids and organoids were pre-washed with DMEM 10% or Neuronal media, respectively. Freshly egressed tachyzoites (1–5 × 10^4^ cfu/well) or infected DCs (1 × 10^4^ cfu/well) were added and incubated for 4 h, 16–24 h (final volume of 100 µl/well). In co-infection assay, spheroids were simultaneously incubated with RH-GFP tachyzoites (2.5 × 10^4^ cfu/well) and ME49-RFP tachyzoites (2.5 × 10^4^ cfu/well) for 24 h. After infection, spheroids and organoids were washed in PBS, fixed in 4% paraformaldehyde (PFA) (Histolab) for 3 h at 4 °C, stained with DAPI (Merk) or methyl green (Thermo Scientific) and imaged using a LSM 800 Airyscan, Zeiss confocal microscope.

### Primary DCs, macrophages and* T. gondii *challenge

Murine bone marrow-derived DCs [61] and macrophages [30] were generated as previously described. Bone marrow cells were purified from 6 to 8-week-old male or female mice and cultured in RPMI 1640 medium (VWR) supplemented with 10% FBS, 20 µg/ml gentamicin, 10 mM HEPES and 10 ng/ml recombinant GM-CSF for DCs or macrophages (PeproTech) or M-CSF (Peprotech) for macrophages. DCs (loosely adherent cells), GM-CSF macrophages (adherent cells) and M-CSF macrophages (adherent cells) were harvested after 6 days, pre-labelled with CFSE (green), CMTMR (orange) or CMF2HC (blue) (Invitrogen) and challenged with freshly egressed *T. gondii* tachyzoites at multiplicity of infection (MOI) 1 (type I, RH) or MOI 2 (type II, ME49, PRU) for 4 h to obtain an infection frequency of ∼50%. Cell suspensions were then washed twice and spun at 60xg to minimize free tachyzoites in the culture. Total numbers of colony-forming units (cfu) were confirmed by plaquing assays. Host cell number and viability was assessed by ocular hemocytometry and flow cytometry, respectively.

### Inhibition of motility and adhesion

CMTMR or CM2FHC pre-labelled DCs were infected with type II PRU-GFP tachyzoites (MOI 2), as described above. Infected DCs (2 × 10^5^/ml) were incubated in RPMI 10% media with 20mM ROCK inhibitor Y-27,632 (Merk), 2 mM GM6001 MMP inhibitor (Merk), DMSO (Merk), 1 µg/ml anti-mo CD54 (ICAM-1) (eBioscience) or 1 µg/ml isotype antibody (eBioscience) at 37 °C, 5% CO2 for 1 h. Treated DCs were then incubated with spheroids (1 × 10^4^/spheroid) in presence of inhibitors or antibodies for 16 h. Alternatively, infected DCs were resuspended in RPMI 10% containing 500 U/ml heparin (Sigma) or control media, immediately transferred to spheroids and incubated for 16 h. Then, samples were washed in PBS, fixed in PFA 4%, stained with methyl green (nuclei) and imaged using a LSM 800 Airyscan, Zeiss confocal microscope.

### Immunohistochemistry and immunostainings

For bEnd.3 spheroids and organoids, ∼50 constructs were collected in 1.5 ml tubes, washed in PBS, and fixed in 4% PFA for 3 h at 4 °C. Spheroids/organoids were permeabilized in PBS with 1% Triton X-100 (Sigma) for 1 h at RT, dehydrated through a methanol (Fisher Chemical) gradient (25%, 50%, 75%, 100%), and incubated in blocking buffer (PBS with 0.1% Triton X-100 and 5% FBS) at 4 °C for 16 h. Then, samples were incubated with primary antibodies, anti ZO-1 (Invitrogen), anti-CD31/PECAM-1 (BD Biosciences), anti-GLUT-1 (Santa Cruz Biotechnology), anti-Map2 (Abcam), anti-GFAP (Invitrogen,) rabbit polyclonal anti-GRA7 (a gift from E. Linder, Statens Bakteriologiska Laboratorium, Solna, Sweden) [62] diluted 1/250 (v/v) in blocking buffer, at 4 °C for 48 h. After washing, samples were incubated with Alexa Fluor-conjugated secondary antibodies for 24 h at 4 °C, washed, stained with DAPI (30 min), mounted in PBS using culture well gaskets (Grace Bio-Labs) and imaged with a Zeiss LSM 800 confocal microscope. Signal with mab anti-ZO1/Alexa Fluor 647 was noted on parasite vacuoles within spheroids. This signal was similarly present in vacuoles in HFF cells, indicating cross-reactivity of antibodies, but had no impact on localization analyses or calculations of migrated distances in spheroids.

For bEnd.3 monolayers, cells (5 × 10^4^/well) were seeded onto cover glasses in 24-well plates. After reaching 100% confluence at day 3 post seeding, media was replaced by fresh DMEM 10% and cells incubated for 24 h. Cells were washed 3 times in PBS and fixed in 4% PFA for 10 min at RT, permeabilized in PBS with 0.1% Triton X-100 for 15 min at RT, washed in PBS and incubated in blocking buffer (PBS with 0.1% Triton X-100 and 5% FBS) for 1 h at RT. Then, samples were incubated with primary antbodies anti-ZO-1 (Invitrogen), anti-CD31/PECAM-1 (BD Biosciences) and anti-GLUT-1 (Santa Cruz Biotechnology), diluted 1:500 (v/v) in blocking buffer, at 4 °C for 24 h. After washing in PBS 0.1% Triton X-100, samples were incubated with Alexa-conjugated secondary antibodies (ThermoFisher) for 1 h at RT, washed in PBS, and imaged with Zeiss LSM 800 confocal microscope.

### Image acquisition and processing

To quantify the localization of *T. gondii* tachyzoites and infected DCs, spheroids/organoids were imaged using confocal microscopy (LSM 800 Airyscan, Zeiss) with laser lines 405 (for DAPI and CM2FHC) 488 nm (for GFP and CFSE), 561 nm (for RFP, CMTMR and Alexa 594) and 640 nm (For Evans blue, Alexa 647 and methyl green). Z-stacks (0 to 70 μm in depth, 1 μm interval) were collected using a 40x objective. Images were processed with Imaris v.10.1 software, using the *Surface* rendering tool to semi-automatically define the spheroid surface, and the *Spots* rendering tool to automatically identified GFP/RFP-expressing tachyzoites and pre-labeled phagocytes (DCs, macrophages). Euclidian distances to the spheroid/organoid surface were calculated using *Shortest Distance To Surfaces* tool. Spots were colored with *Statistical Color-Coding* tool choosing the parameter *Distance To Surface*. Data tables were exported and plotted with GraphPad Prism v.10 software. The procedures for 3D Surfaces analyses of Euclidian distances migrated by tachyzoites and infected DCs in spheroids and organoids are detailed in Fig S2. *Diffraction PSF 3D* and *Interactive Deconvolve 3D* plugins from FIJI/ImageJ software were used for deconvolution of micrographs in Figs. 3 and 6.

### Statistical analyses

All statistical analyses were performed using GraphPad Prism v. 10. For comparisons among multiple groups, one-way ANOVA followed Bonferroni’s multiple comparison test were applied on data sets with normal distribution, whereas Kruskal-Wallis test followed Dunn’s post-hoc test was performed on non-normal distributed data sets. For comparisons between 2 groups with normal distribution, two-tailed Student’s t-test was applied. For comparisons between 2 groups with non-normal distribution, Mann-Whitney U-test was applied. A p-value < 0.05 was defined as significant differences for all statistical tests.

## Supplementary Information

Below is the link to the electronic supplementary material.

Supplementary Material 1 (Movie S1 MP4 43.4 MB)

Supplementary Material 2 (Movie S2 MP4 17.3 MB)

Supplementary Material 3 (Movie S3 MP4 3.94 MB)

Supplementary Material 4 (Movie S4 MP4 8.03 MB)

Supplementary Material 5 (Movie S5 MP4 4.77 MB)

Supplementary Material 6 (Movie S6 MP4 50.5 MB)

Supplementary Material 7 ( Movie S1-S6 legends PDF 30.9 KB)

Supplementary Material 8 (Table S1, Figs S1-S4 PDF 2.37 MB)

## Data Availability

The datasets generated during and/or analyzed during the current study are available from the corresponding author on reasonable request.
