# Supplementary material for: Brain endothelial spheroids and cortical organoids reveal the impact of Toxoplasma gondii lineage and host-phagocyte-pathogen interactions on colonization
Source: Cell Mol Life Sci. 2026 Jan 29;83(1):86. doi: 10.1007/s00018-025-06035-7 (PMC12860780; doi:10.1007/s00018-025-06035-7)
Supplement: Supplementary file 7 — Supplementary Material 7 ( Movie S1-S6 legends PDF 30.9 KB) [file 18_2025_6035_MOESM7_ESM.pdf]

Supplementary material ESM 1

Brain endothelial spheroids and cortical organoids reveal the impact of *Toxoplasma gondii* lineage and host-phagocyte-pathogen interactions on colonization

Matias E. Rodriguez[1], Elena Afanaseva[1], Ali Hassan[1], Felix Harryson-Oliveberg[1], Antonio Barragan[1, *]

[1]Department of Molecular Biosciences, The Wenner-Gren Institute, Stockholm University, Stockholm, Sweden

* Correspondence: antonio.barragan@su.se

Movie S1-S6 legends

Movie S1. Generation of 3D maps from b.End3 spheroids challenged with *T. gondii* tachyzoites

Spheroids were challenged with 5x10^4 cfu type I RH (Tg I, GFP$^+$, green) tachyzoites. After 16 h, spheroids were fixed and nuclei stained (DAPI$^+$, blue). Movie shows 3D reconstruction from confocal image stack generated from the surface to 70 µm depth as indicated under Materials and Methods. Individual GFP$^+$ tachyzoites were color-coded by distance to the surface. Color scale 0 to 40 µm. Scale bar: 30 µm *(mp4).*

Movie S2. 3D map of b.End3 spheroids challenged with *T. gondii* (Tg I)

Spheroids were challenged with 5x10^4 cfu type I RH-GFP tachyzoites and analyzed as indicated in Movie S1. Color scale: 0 to 40 µm depth. Scale bar: 30 µm *(mp4).*

Movie S3. 3D map of b.End3 spheroids challenged with *T. gondii* (Tg II)

Spheroids were challenged with 5x10^4 cfu type II ME49-RFP tachyzoites and analyzed as indicated in Movie S1. Color scale: 0 to 40 µm depth. Scale bar: 30 µm *(mp4).*

Movie S4. 3D reconstruction of an infected DC infiltrating a b.End3 spheroid.

CMTMR pre-labelled DCs were challenged with *T. gondii* (RH-GFP, MOI 1) to obtain a DC infection frequency of ~50%. Then, spheroids challenged with *T. gondii* (green)-infected DCs (red) (2x10^4 DCs / ~1x10^4 cfu *Tg*/spheroid). After 16 h, spheroid was fixed and nuclei stained (DAPI$^+$, blue) as indicated under Materials and Methods. Movie shows close-up and 3D reconstruction of a parasitized DC with an extended membrane protrusion infiltrating layers of the spheroid. Scale bar: 15 µm *(mp4).*

Movie S5. Confocal stacks of infected DCs infiltrating a b.End3 spheroid.

CMTMR pre-labelled DCs were challenged with *T. gondii* (RH-GFP, MOI 1) to obtain a DC infection frequency of ~50%. Then, spheroids challenged with *T. gondii* (green)-infected DCs (red) (2x10^4 DCs / ~1x10^4 cfu *Tg*/spheroid). After 16 h, spheroid was fixed, nuclei stained (DAPI$^+$, blue) and imaged as indicated under Materials and Methods. Confocal stack (0-70 µm depth) with 1 µm interval distance between images is shown. Scale bar: 30 µm *(mp4).*

Movie S6. Constructs with mixed b.End3 cell and cortex-derived cell suspensions

Cell suspensions of CFSE-prelabeled bEnd.3 endothelial cells (green), CMHC-prelabeled astrocytes (blue) and CMTMR-prelabeled cortical cells (red) were mixed and plated. After 3 days, cells were fixed and imaged as indicated under Materials and Methods. 3D reconstruction illustrates how mixed cell suspensions organize into juxtaposed b.End3 spheroids (CFSE+, green) and a cortical cell organoids (cyan, red). Scale bar: 30 µm *(mp4).*
