# Supplementary material for: Brain endothelial spheroids and cortical organoids reveal the impact of Toxoplasma gondii lineage and host-phagocyte-pathogen interactions on colonization
Source: Cell Mol Life Sci. 2026 Jan 29;83(1):86. doi: 10.1007/s00018-025-06035-7 (PMC12860780; doi:10.1007/s00018-025-06035-7)
Supplement: Supplementary file 8 — Supplementary Material 8 (Table S1, Figs S1-S4 PDF 2.37 MB) [file 18_2025_6035_MOESM8_ESM.pdf]

Supplementary material ESM 2

Table S1

Figures S1-S4

Brain endothelial spheroids and cortical organoids reveal the impact of *Toxoplasma gondii* lineage and host-phagocyte-pathogen interactions on colonization

Matias E. Rodriguez[1], Elena Afanaseva[1], Ali Hassan[1], Felix Harryson-Oliveberg[1], Antonio Barragan[1,]*

[1]Department of Molecular Biosciences, The Wenner-Gren Institute, Stockholm University, Stockholm, Sweden

* Correspondence: antonio.barragan@su.se

Table S1. Primers used in this study

Name	Target gene	Sequence
mOccludin_Fw	*Ocln*	TGGCAAGCGATCATACCCAGAG
mOccludin_Rev	*Ocln*	CTGCCTGAAGTCATCCACACTC
mPecam1_Fw	*Pecam 1*	CCAAAGCCAGTAGCATCATGGTC
mPecam1_rev	*Pecam 1*	GGATGGTGAAGTTGGCTACAGG
mCadherin 5_Fw	*Cdh 5*	GAACGAGGACAGCAACTTCACC
mCadherin 5_Rev	*Cdn 5*	GTTAGCGTGCTGGTTCCAGTCA
mPgp1_Fw	*Pgp-1*	TCCTCACCAAGCGACTCCGATA
mPgp1_Rev	*Pgp-1*	ACTTGAGCAGCATCGTTGGCGA
mGlut-1_Fw	*Slc2a1*	GCTTCTCCAACTGGACCTCAAAC
mGlut-1_Rev	*Slc2a1*	ACGAGGAGCACCGTGAAGATGA
mGAPDH_Fw	*gapdh*	TGACCTCAA CTACATGGTCTACA
mGAPDH_Rev	*gapdh*	CTTCCCATT CTCGGCCTTG
mZO1_Fw	*Tjp 1*	GACCTTGATTTGCATGACGA
mZO1_Rev	*Tjp 1*	AGGACCGTGTAATGGCAGAC
mClaudin2_fw	*Cldn2*	ATACTACCCTTTAGCCCTGACCGAGA
mClaudin2_Rev	*Cldn2*	CAGTAGGAGCACACATAACAGCTACC

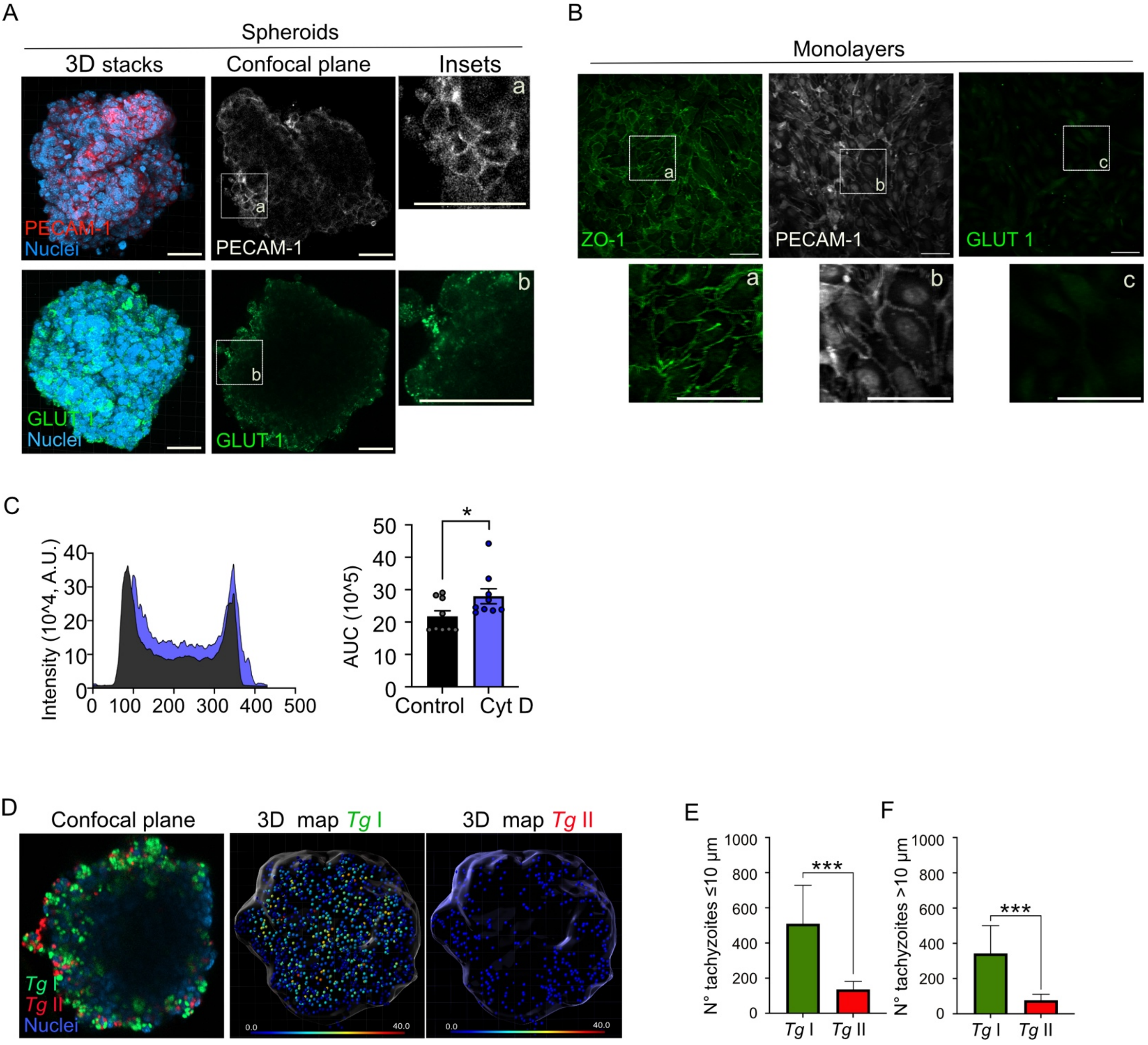

Figure S1. Characteristics of *T. gondii* colonization of spheroids

A. 3D projection (left) and confocal plane (right) micrographs show the localization of nuclei (DAPI$^+$, blue), PECAM-1 (red) and GLUT 1 (green) in b.End3 spheroid. b.End3 cells (5x10^3/well) were seeded into U-bottom non-adherent plates and incubated for 72 h. Spheroids (1/well) were fixed and stained with indicated antibodies. Scale bars, 50 µm.

B. Confocal micrographs show the localization of ZO-1 (green), PECAM-1 (grey) and GLUT 1 (green) in b.End3 monolayers. b.End3 cells (5x10^4/well) were seeded into 24-well plate for 4 days. Monolayers were fixed and stained with indicated antibodies. Scale bar, 50 µm.

C. Plot shows the mean relative fluorescence intensity (arbitrary units, A. U.) of Evans blue (EB) across the spheroid midline. Bar graph shows area-under-the curve analysis. (n = 8 spheroids) from 3 independent experiments.

D. Confocal micrographs (left) show localization of type I RH (Tg I, GFP^{+}, green) and type II ME49 (Tg II, RFP^{+}, red) tachyzoites in relation to nuclei ($DAPI^{+}$, blue) at 50 µm depth inside spheroids following co-incubation with $2{,}5x10^{4}$ Tg I + $2{,}5x10^{4}$ Tg II cfu *Tg*/spheroid for 24 h. Scale bars: 50 µm. 3D surface analysis (right) from confocal stacks (0-70 µm depth) show the 3D distribution of Tg I and Tg II tachyzoites in the same spheroid. The 3D maps show the surface of the spheroids and the tachyzoites, represented by the dots, color-coded by their distance from the nearest surface (µm).

E, F. Graphs show the absolute numbers (mean ± SEM) of type I RH-GFP and type II Me49-RFP tachyzoite per spheroids at ≤ 10 µm (surface, G) and > 10 µm (deeper layers, H), following co-incubation with $2{,}5x10^{4}$ RH + $2{,}5x10^{4}$ ME49 cfu *Tg*/spheroid for 24 h (n = 8 spheroids/condition) from 3 independent experiments.

(C, E, F): 2-tailed unpaired Student's *t*-test; * $p < 0.05$; *** $p < 0.001$.

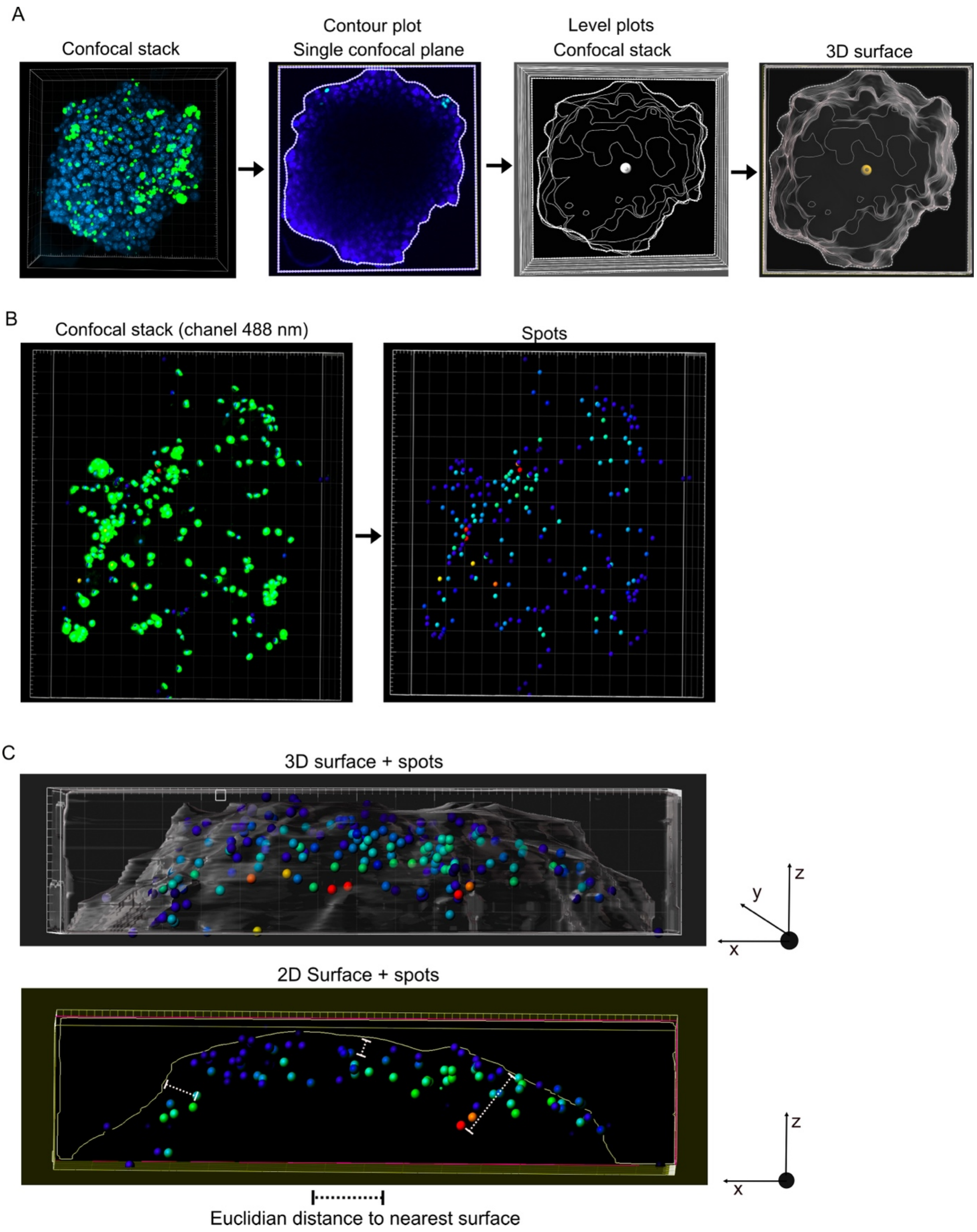

Fig. S2. Measurements of migrated distances in spheroids and organoids

Images show undertaken steps to determine migrated Euclidian distances by GFP/RFP-expressing extracellular *T. gondii* tachyzoites (type I and II) and pre-labeled tachyzoite-infected phagocytes (DCs, macrophages) in spheroids and organoids.

A. 3D surface generation. From a confocal micrograph stack, contour plots of confocal planes were first generated, typically 7-10 per spheroid or organoid (0-70 μm). Then, level curves were generated. Based on level curves, a 3D surface plot was created using the *Surface* rendering tool (Imaris v.10.1 software) to semi-automatically define the spheroid surface.

B. 3D spot generation. From a confocal micrograph stack, spots were automatically generated using the *Spots* rendering tool to automatically identify GFP/RFP-expressing tachyzoites. When indicated, the same tool was applied to identify pre-labeled phagocytes (DCs, macrophages). Size filtration was applied if needed.

C. Distances to the spheroid/organoid surface. Euclidian distances to the spheroid/organoid surface were calculated using *Shortest Distance To Surfaces* tool and numeric data table exported to GraphPad software (v.10). Spots were colored with *Statistical Color-Coding* tool choosing the parameter *Distance To Surface*. White doted lines illustrate Euclidian distance to surface.

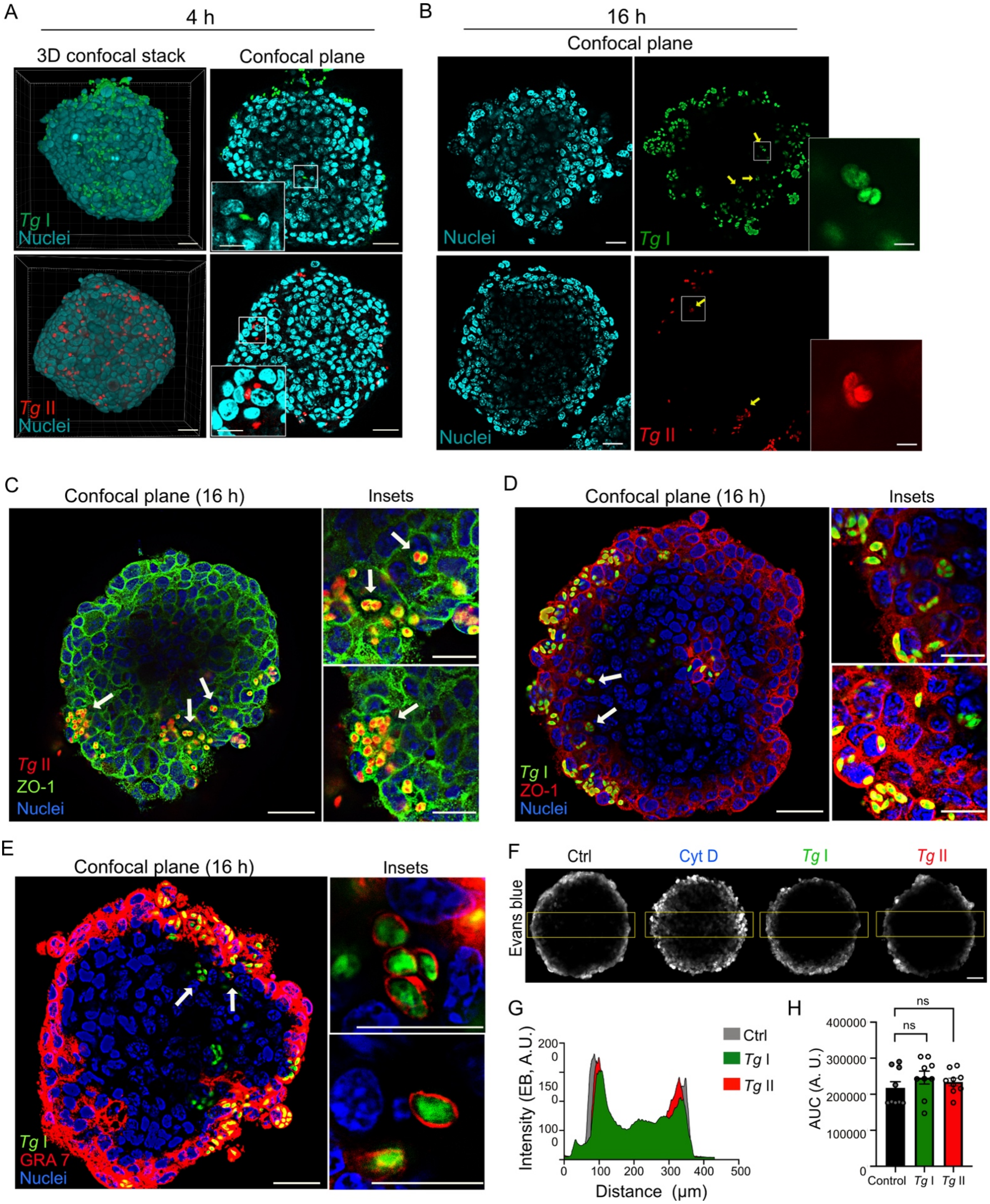

Fig S3. **Kinetics of transmigration and effects of *T. gondii* on the permeability of spheroids**

A. Confocal micrographs show 3D stack and confocal plane at 50 µm depth, with localization of type I RH (*Tg* I, GFP^{+}, green) and type II ME49 (Tg II, RFP^{+}, red) tachyzoites in relation to nuclei (DAPI^{+}, cyan) inside spheroids following incubation with 2x10^{5} cfu *Tg*/spheroid for 4 h.

Insets show magnifications of individual (non-replicated) *Tg* I and *Tg* II tachyzoites in inner cellular layers. Scale bars: 50 μm; insets 10 μm.

B. Confocal micrographs show single plane at 50 μm depth, with conditions as in (A) except challenge with $5x10^4$ cfu *Tg*/spheroid at 16 h timepoint. Arrows indicate loci in inner cellular layers with replicating parasites and are magnified in the insets *Tg* I and *Tg* II, respectively. Scale bars: 50 μm; insets 10 μm.

C. Deconvolved confocal micrograph shows localization of type II ME49 tachyzoites (RFP^+, red), ZO-1 (anti-ZO-1/ Alexa 647, green) and nuclei ($DAPI^+$, blue) at 50 μm plane depth in spheroids following incubation with $5x10^4$ cfu *Tg*/spheroid for 16 h. Arrows indicate loci in inner cellular layers with replicating parasites, magnified in the insets. Scale bars: 30 μm, inset scale bar: 15 μm.

D. Deconvolved confocal micrograph shows localization of type I RH tachyzoites (GFP^+, green), ZO-1 (anti-ZO-1/ Alexa 647, red) and nuclei ($DAPI^+$, blue) at 50 μm plane depth in spheroids following incubation with $5x10^4$ cfu *Tg*/spheroid for 16 h. Arrows indicate loci in inner cellular layers with replicating parasites, magnified in the insets. Scale bars: 30 μm, inset scale bar: 15 μm.

E. Deconvolved confocal micrograph shows localization of type I RH tachyzoites (GFP^+, green), GRA7 (anti-GRA7/ Alexa 647, red) and nuclei ($DAPI^+$, blue) at 50 μm plane depth in spheroids following incubation with $5x10^4$ cfu *Tg*/spheroid for 16 h. Arrows indicate loci in inner cellular layers with replicating parasites (GFP^+) located in PVs ($GRA7^+$), and are magnified in the insets, respectively. Scale bars: 30 μm, inset scale bar: 15 μm.

F. Spheroids were challenged with type I or type II tachyzoites ($2x10^5$ cfu *Tg*/spheroid) or control (Ctrl) media for 4 h, following incubation with Evans blue (EB) +/- cytochalasin D (Cyt D) for 1h. Confocal micrographs show fluorescence signal of EB (grey). Yellow rectangles show sampled areas for fluorescence intensity analyses (shown in Fig. 2D). Scale bar, 50 μm.

G. Plot shows the mean relative fluorescence intensity (arbitrary units, A. U.) of Evans blue (EB) across the spheroid midline as in (E). For each condition, color-coded curves show mean signal from 9 separate spheroids from 2 independent experiments.

H. Bar graph shows area-under-the curve analysis of mean relative fluorescence intensity (arbitrary units, A. U.) of Evans blue (EB) across the spheroid midline of 9 spheroids per condition, as in (F). (n = 9 spheroids/condition) from 2 independent experiments. One-way ANOVA followed by Bonferroni's multiple comparison test. ns: non-significant.

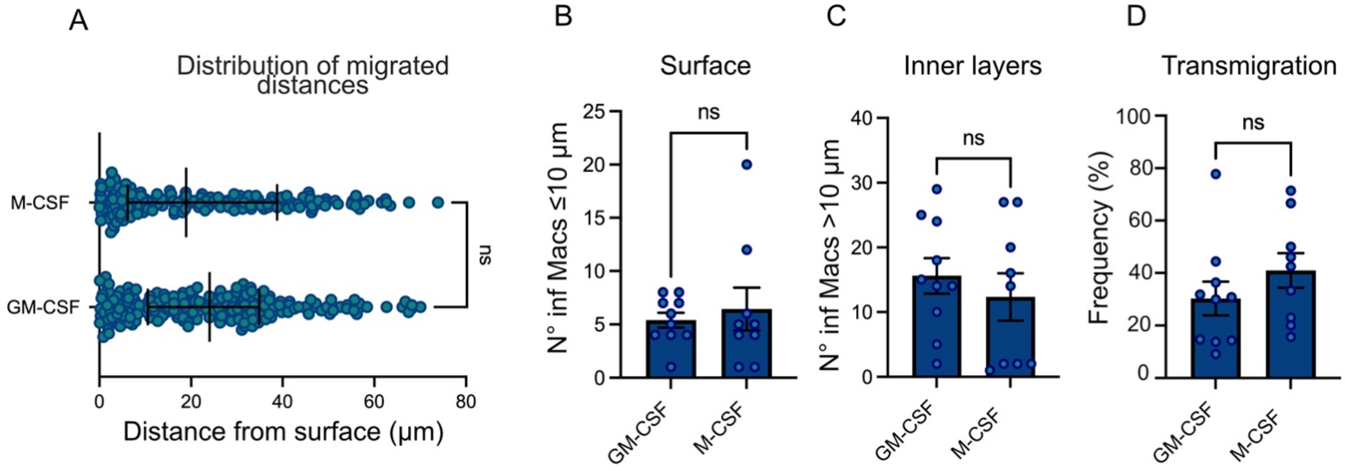

Fig S4. Colonization of spheroids by *T. gondii*-infected macrophages

A. Graph shows the distribution of migrated distances by M-CSF or GM-CSF-derived macrophages (Macs) challenged with type II (PRU-GFP) tachyzoites (2x10^4 Macs / ~1x10^4 cfu *Tg*/spheroid) for 16 h. Median, 25th and 75th percentiles are shown (n = 174 (M-CSF) and 201 (GM-CSF) infected Macs from 9-10 spheroids/condition from 3 independent experiments).

B, C. Graphs show the absolute numbers (mean ± SEM) of infected Macs per spheroid located ≤ 10 µm from the surface (B) and > 10 µm (C, inner layers) following incubation with 2x10^4 Macs / ~1x10^4 cfu *Tg*/spheroid for 16 h. (n = 9-10 spheroids/condition).

D. Graph shows the percentage (mean ± SEM) of infected Macs penetrating >10 µm (transmigration) related to total numbers in spheroids, following incubation with 2x10^4 Macs / ~1x104 cfu Tg/spheroid for 16 h. (n = 9-10 spheroids/condition).

All data are from 3 independent experiments. (A): 2-tailed Mann-Whitney U-test; (B, C, D): 2-tailed unpaired Student's t-test. ns: non-significant.
